# Biochar in Animal Agriculture: Enhancing Health, Efficiency and Environmental Sustainability

**DOI:** 10.1002/vms3.70629

**Published:** 2025-10-14

**Authors:** Mohsen Kazemi

**Affiliations:** ^1^ Department of Animal Science Faculty of Agriculture and Animal Science University of Torbat‐e Jam Torbat‐e Jam Iran

**Keywords:** biochar, circular economy, livestock health, methane reduction, sustainable agriculture

## Abstract

Biochar, a carbon‐rich material produced through the pyrolysis of organic biomass (e.g., wood, crop residues, manure and other organic wastes), has emerged ‐as an effective solution for enhancing sustainability in animal agriculture. This article reviews and integrates current research on biochar's multifaceted roles, including its use as a feed additive to improve gut health, nutrient absorption and growth performance. Notably, innovative applications of biochar in enhancing feed formulations and promoting animal resilience against diseases are discussed. Recent studies have shown that biochar can also enhance animal resilience against diseases and promote a healthier gut microbiome, which is essential for overall livestock productivity and well‐being. Moreover, its unique properties allow for the development of biochar‐based products that can significantly reduce feed costs while improving overall animal health. Additionally, biochar has been linked to improved feed efficiency, leading to better weight gain and reduced feed costs. Its ability to mitigate environmental impacts by reducing methane emissions and ammonia volatilization in manure management, and its long‐term carbon sequestration potential are significant. Furthermore, the integration of biochar into circular agricultural systems is explored, highlighting its role in transforming waste into valuable resources. Moreover, biochar contributes to soil health by improving nutrient retention and water‐holding capacity, which is crucial for sustainable farming practices. This enhancement leads to increased crop yields and a reduction in the reliance on synthetic fertilizers, thereby promoting a more circular agricultural economy. Despite these benefits, challenges such as variability in biochar quality, economic feasibility and the ‐need for standardized guidelines remain. Addressing these challenges is essential for widespread adoption and effective use in various agricultural systems, ensuring that biochar can be safely integrated into existing farming practices. Furthermore, this article underscores biochar's potential to bridge productivity and ecological sustainability, while calling for further research to optimize its applications and ensure safe, large‐scale implementation in diverse livestock production systems.

## Graphical abstract

Biochar enhances animal agriculture by improving gut health (8%–26% higher feed efficiency), reducing methane emissions (up to 22%) and ammonia volatilization (40%–77%) and sequestering carbon. Challenges include variability in quality and economic feasibility, but its integration offers a sustainable path for productivity and environmental stewardship.

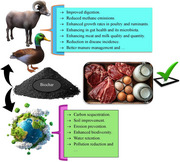

## 1 Introduction

The global demand for animal‐derived products, such as meat, milk and eggs, has been steadily increasing due to population growth, urbanization and rising incomes. This surge in demand has placed immense pressure on livestock and poultry production systems to enhance productivity while addressing environmental, economic and ethical challenges. Traditional animal farming practices often rely on intensive methods that can lead to issues such as environmental pollution, resource depletion and animal health concerns (Schmidt et al. 2019). In this context, there is a growing need for innovative and sustainable solutions to improve the efficiency and sustainability of animal production. One such solution that has garnered significant attention in recent years is the use of biochar. Biochar, a carbon‐rich material produced through the pyrolysis of organic biomass under limited oxygen conditions, has been widely recognized for its potential applications in agriculture, environmental management and animal nutrition (Chandrasekharan et al. 2024; Waheed et al. 2025; Hegarty et al. 2024). Historically, it has been used for soil amendment, particularly in the context of improving soil fertility and carbon sequestration (Tsolis and Barouchas 2023; Nepal et al. 2023). However, its applications have expanded beyond agriculture, with emerging evidence suggesting its potential benefits in animal production systems. The unique physicochemical properties of biochar, such as its high surface area, porosity and adsorption capacity, make it a versatile tool for addressing various challenges in livestock and poultry farming (Leng et al. 2021; Wang et al. 2024). The use of biochar in animal production has historical roots in indigenous agricultural systems. Documented practices include the Amazonian terra preta tradition, where biochar‐amended soils improved forage quality for grazing animals, along with Japanese beka‐bokashi methods that incorporated charcoal into poultry feed for improved digestion. Similar applications have been observed among West African pastoralists who traditionally used charcoal to treat ruminant digestive disorders. These cross‐cultural examples demonstrate that the benefits of carbon‐rich amendments in animal husbandry have been empirically recognized for centuries, long before modern scientific validation. However, modern scientific research has only recently begun to explore the mechanisms and benefits of biochar in a systematic manner. Studies have shown that biochar can serve as a feed additive, improve gut health, enhance nutrient absorption and reduce the environmental impact of animal farming (Nair et al. 2023; Burezq and Khalil 2025). These findings have generated significant interest among researchers, farmers and policymakers, who are increasingly recognizing biochar as a multifunctional tool for promoting sustainable animal production. One of the primary applications of biochar in livestock and poultry production is its use as a feed additive. When added to animal diets, biochar has been shown to improve growth performance, feed efficiency and overall animal health (Schmidt et al. 2019; Konduri et al. 2024). The porous structure of biochar allows it to adsorb toxins, pathogens and harmful gases in the digestive tract, thereby reducing the risk of diseases and enhancing nutrient utilization (Toth and Dou 2016; O'Toole et al. 2016; Mukherjee et al. 2022). Additionally, the separate use of probiotics, specifically Bacilli and Lactobacilli, or biochar in the diet enhanced in vitro ruminal microbial and enzymatic activity while reducing energy losses (as methane) and nitrogen losses (as ammonia) (Bagherpoor et al. 2023). Notably, adding biochar to probiotic‐containing diets proved to be an effective strategy for improving digestibility and microbial biomass and reducing methane and ammonia (Bagherpoor et al. 2023). Another significant application of biochar is in manure management (Sánchez‐Monedero et al. 2019; Chung et al. 2021). Livestock and poultry farming generate large quantities of manure, which, if not managed properly, can lead to environmental pollution and public health concerns. Because of the high nutrient content, chicken manure is widely used as a fertilizer in agriculture (Ravindran et al. 2017; Won et al. 2018). However, the direct use of untreated chicken manure poses significant environmental risks, including eutrophication, greenhouse gas emissions, contamination of air and water resources, release of phytotoxic compounds and potential threats to human health through disease transmission and foodborne pathogens. As a result, chicken manure should be treated and managed properly before its application as a biofertilizer in agricultural farmland (Li et al. 2020; Ravindran et al. 2017). The study shows that adding biochar to chicken manure compost reduces harmful gas emissions and pathogens, while also improving the compost's nutrient content (Chung et al. 2021). By incorporating biochar into manure management practices, farmers can mitigate the environmental impact of animal farming while establishing a closed‐loop system. This circular approach transforms agricultural waste into valuable resources: Biochar derived from crop residues is used to treat livestock manure, which is subsequently reapplied to fields as enriched organic fertilizer. Such systems optimize resource efficiency by completing nutrient cycles and reducing dependence on external inputs. The environmental benefits of biochar extend beyond manure management. Biochar is known for its ability to sequester carbon, thereby contributing to climate change mitigation (Lorenz and Lal 2014). When applied to soils or used in animal production, biochar can reduce greenhouse gas emissions, such as methane and nitrous oxide, which are associated with livestock farming (Chang et al. 2016; Kammann et al. 2017; Shrestha et al. 2023). Furthermore, the production of biochar from agricultural waste materials provides an opportunity to convert low‐value biomass into a high‐value product, reducing waste and promoting circular economy principles (Pavesi et al. 2025). Despite its potential benefits, the use of biochar in livestock and poultry production is not without challenges. The quality and composition of biochar can vary significantly depending on the feedstock and pyrolysis conditions, which may affect its performance and safety (Tomczyk et al. 2020). There are also concerns about the potential risks of biochar, such as the presence of contaminants (e.g., heavy metals, polycyclic aromatic hydrocarbons and dioxins) or its impact on soil and animal health in the long term (Ndirangu et al. 2019). Additionally, when introduced into the soil, certain types of biochar can release contaminants, which may present risks to both soil and aquatic ecosystems. The heavy metals that are released can be harmful to animals, plants and microorganisms, and they have the potential to accumulate in the food web, endangering human health (Dong et al. 2025). Additionally, the economic feasibility of large‐scale biochar production and application remains a topic of debate, particularly in resource‐limited settings (Keske et al. 2019). Addressing these challenges requires further research, standardization and the development of clear guidelines for biochar use in animal production. The growing body of research on biochar in livestock and poultry production highlights its potential as a sustainable and multifunctional tool. However, there is still much to learn about its mechanisms of action, optimal application methods and long‐term effects on animal health and the environment. The integration of biochar into livestock and poultry production systems offers a promising pathway to address some of the most pressing challenges in animal farming, including environmental pollution, resource inefficiency and animal health concerns. As the global demand for animal‐derived products continues to rise, the adoption of sustainable practices such as biochar application will be critical for ensuring food security, protecting the environment and promoting the well‐being of both animals and humans. To address these critical aspects, this article explores biochar's multifaceted role in animal agriculture through three key dimensions, as presented in Section 1.1.

### 1.1 Goals

This narrative article explores the multifaceted role of biochar in animal agriculture through three key perspectives. First, it describes the fundamental physicochemical characteristics of biochar and explains how these properties influence its effectiveness in livestock production systems. In contrast to previous reviews, this article emphasizes innovative applications of biochar specifically tailored for animal nutrition, showcasing recent advancements that enhance its functional properties. Second, it presents a comprehensive overview of biochar's impacts on animal health and product quality, detailing its effects on digestive processes, disease resistance, growth performance and the nutritional value of meat, milk and eggs. This review further highlights the unique benefits of biochar in promoting gut health through targeted feed formulations, which were less emphasized in earlier literature. Third, the article documents biochar's environmental contributions, particularly its ability to improve manure management, reduce harmful emissions and enhance carbon storage in agricultural systems. Unlike prior reviews that primarily addressed biochar's general environmental benefits, this article integrates recent findings that illustrate its role in fostering a circular economy within livestock systems. Additionally, the discussion addresses practical challenges currently limiting widespread biochar adoption, including product variability, economic considerations and safety assessments. The article concludes by identifying promising research directions that could optimize biochar applications for more sustainable livestock production. By providing a comprehensive synthesis of both foundational research and innovative applications, this narrative aims to equip readers with a thorough understanding of biochar's potential to harmonize agricultural productivity with ecological stewardship.

## 2 Methodology

### 2.1 Literature Search Strategy

This narrative review employed a systematic approach to identify relevant studies through comprehensive searches of multiple academic databases. We queried Springer, Taylor and Francis, Science Direct, PubMed, Scopus, Web of Science, Google Scholar and other relevant sources. Using carefully selected keywords, including ‘biochar’, ‘livestock feed’, ‘animal nutrition’, ‘manure treatment’ and ‘carbon sequestration’. The literature search encompassed publications from 1998 to 2025, with approximately 70% of the references published between 2020 and 2025. This temporal scope was selected to capture both foundational research and recent advancements in biochar applications for animal agriculture. The period reflects key scientific milestones, including (1) the growing recognition of biochar's role in carbon sequestration, (2) its demonstrated efficacy in soil health improvement and (3) emerging evidence of its benefits for livestock nutrition and environmental sustainability.

### 2.2 Study Selection Criteria

The review established clear inclusion and exclusion criteria to ensure the relevance and quality of selected studies. Peer‐reviewed original research articles, review papers and meta‐analyses that specifically investigated biochar's effects on animal health, production parameters or environmental impacts in agricultural systems were included. Excluded were non‐peer‐reviewed publications, studies unrelated to livestock production and duplicate reports, resulting in high‐quality studies for evaluation. Additionally, only studies published in English were considered to maintain consistency and comprehensibility in the analysis. This approach ensured that all selected studies met rigorous academic standards and were accessible for thorough review.

### 2.3 Data Extraction and Organization

A structured data extraction process was implemented to systematically categorize findings into three primary thematic areas. The first category examined biochar's physicochemical characteristics, including production methods and material properties. The second focused on animal performance metrics, such as growth rates, feed efficiency and product quality. The third analysed environmental outcomes, including manure management efficiency and greenhouse gas mitigation potential.

### 2.4 Quality Assessment Framework

Given the narrative nature of this review, the quality assessment focused on evaluating the completeness and clarity of information presented in the selected studies rather than quantitative analysis. All included publications were carefully examined for methodological transparency, with particular attention to

1. Detailed description of biochar production methods (feedstock type, pyrolysis conditions)
2. Clear reporting of experimental designs in animal trials
3. Comprehensive presentation of results and discussion
4. Appropriate interpretation of findings within study limitations

The review incorporated studies across various journal tiers to ensure broad representation of research perspectives, prioritizing those that provided thorough methodological details regardless of journal impact factor. This inclusive approach allowed for a comprehensive synthesis of available knowledge while acknowledging variations in study quality and design. The assessment emphasized how well each study contributed to understanding biochar's multifaceted roles in animal agriculture rather than applying rigid quality scoring systems.

### 2.5 Methodological Limitations

The review acknowledges several inherent limitations in its approach. Potential publication bias may exist due to the greater likelihood of positive results being published. Variability in biochar feedstocks, production methods and application rates across studies presents challenges for direct comparison. Despite these limitations, the methodology provides a robust foundation for the comprehensive narrative synthesis that follows.

## 3 Physicochemical Properties of Biochar

### 3.1 Composition and Structure

**FIGURE 1:**
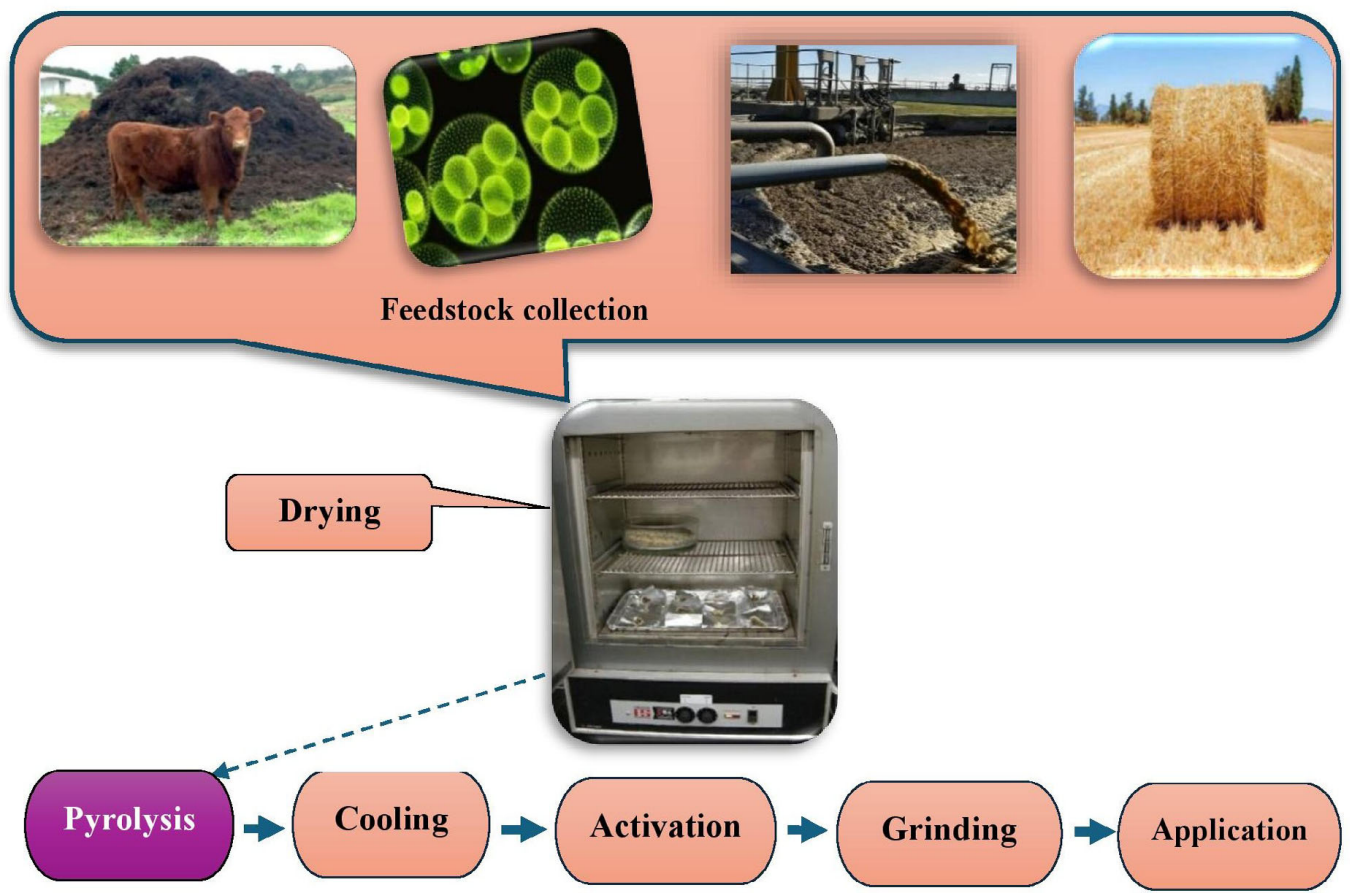
An overview of biochar preparation.

Biochar is a carbon‐rich material derived from the pyrolysis of organic biomass, such as agricultural residues, wood, manure and other organic waste, under limited oxygen conditions (Sri Shalini et al. 2021). Its composition and structure are highly influenced by factors such as feedstock type, pyrolysis temperature, heating rate and duration (Lataf et al. 2022). Generally, biochar consists of a complex matrix of organic carbon, minerals and ash, with carbon content ranging from 50% to 90% depending on the feedstock and pyrolysis conditions (Amalina et al. 2022; Li and Tasnady 2023). The high carbon content makes biochar a stable and recalcitrant material, resistant to microbial degradation and capable of persisting in the environment for hundreds to thousands of years. The structure of biochar is characterized by its highly porous nature, with a large surface area and a network of micro‐, meso‐ and macropores. These pores are formed during the pyrolysis process as volatile compounds are released, leaving behind a rigid carbon framework. The surface area and total pore volume of biochar typically range from 8 to 132 m^2^/g and 0.016 to 0.083 cm^3^/g, respectively (Leng et al. 2021). These values depend on the type of feedstock and the pyrolysis temperature. Higher pyrolysis temperatures generally lead to increased porosity and surface area, which enhances the adsorption capacity of biochar. Moderate temperatures (400–700°C) are ideal for developing the pore structure (Leng et al. 2021). This porous structure not only provides habitat for beneficial microorganisms but also allows biochar to adsorb nutrients, toxins and gases, making it a versatile material for various applications in livestock and poultry production. Furthermore, another method worth considering is hydrothermal carbonization (HTC). This process involves the treatment of biomass with water at elevated temperatures and pressures, leading to the formation of a carbon‐rich material similar to biochar (Satari et al. 2025; Selvaraj et al. 2025). HTC can effectively convert wet biomass, such as agricultural residues and organic waste, into a stable product, thus addressing issues related to feedstock moisture content (Satari et al. 2025; Selvaraj et al. 2025). The resulting biochar from HTC exhibits distinct properties that may enhance its suitability for specific applications in animal agriculture. The biochar produced via HTC acts as a beneficial soil enhancer, boosting fertility and reducing dependence on synthetic fertilizers. This fosters sustainable agriculture by facilitating efficient nutrient reuse. Moreover, HTC transforms low‐value waste into high‐value products, creating economic opportunities in agriculture, clean energy and advanced materials industries. This process aligns with both ecological balance and long‐term economic sustainability (Satari et al. 2025). In addition to carbon, biochar contains essential minerals such as potassium, calcium, magnesium and phosphorus, which can contribute to its nutritional value when used as a feed additive (Schmidt et al. 2019; Vijayaraghavan 2021). The mineral content varies depending on the feedstock; for example, biochar derived from manure or crop residues tends to have higher nutrient concentrations compared to wood‐based biochar. The presence of these minerals can enhance the nutritional profile of animal diets and support metabolic processes. The chemical composition of biochar encompasses various functional groups that influence its reactivity and interactions with other materials. Among these, carboxyl, phenolic hydroxyl, lactone and peroxide groups are the primary oxygen functional groups generated on biochar through the oxidation process (Sajjadi et al. 2019). These groups are crucial for enhancing the biochar's properties and its effectiveness in various applications (Sajjadi et al. 2019). These functional groups enable biochar to participate in ion exchange, chelation and other chemical reactions, further enhancing its ability to adsorb toxins, improve nutrient availability and modulate gut health. Overall, the unique composition and structure of biochar form the basis of its multifunctional properties, making it a valuable tool for improving the sustainability and efficiency of livestock and poultry production systems. An overview of biochar preparation is shown in Figure 1. The figure illustrates that for the production of biochar, raw materials, such as agricultural waste, manure, microalgae or sewage sludge, are first collected and prepared. The biomass is then dried to reduce moisture content, ensuring efficient pyrolysis. Next, the dried biomass undergoes pyrolysis in a low‐oxygen environment at temperatures between 400°C and 700°C, producing biochar, syngas and bio‐oil. The biochar is cooled and optionally activated to enhance its properties. Finally, it is ground or screened to achieve the desired particle size and applied to soil or used in other applications like water filtration and as an additive in animal feed, contributing to improved soil fertility, carbon sequestration and livestock health.

### 3.2 Surface Area and Porosity

**FIGURE 2:**
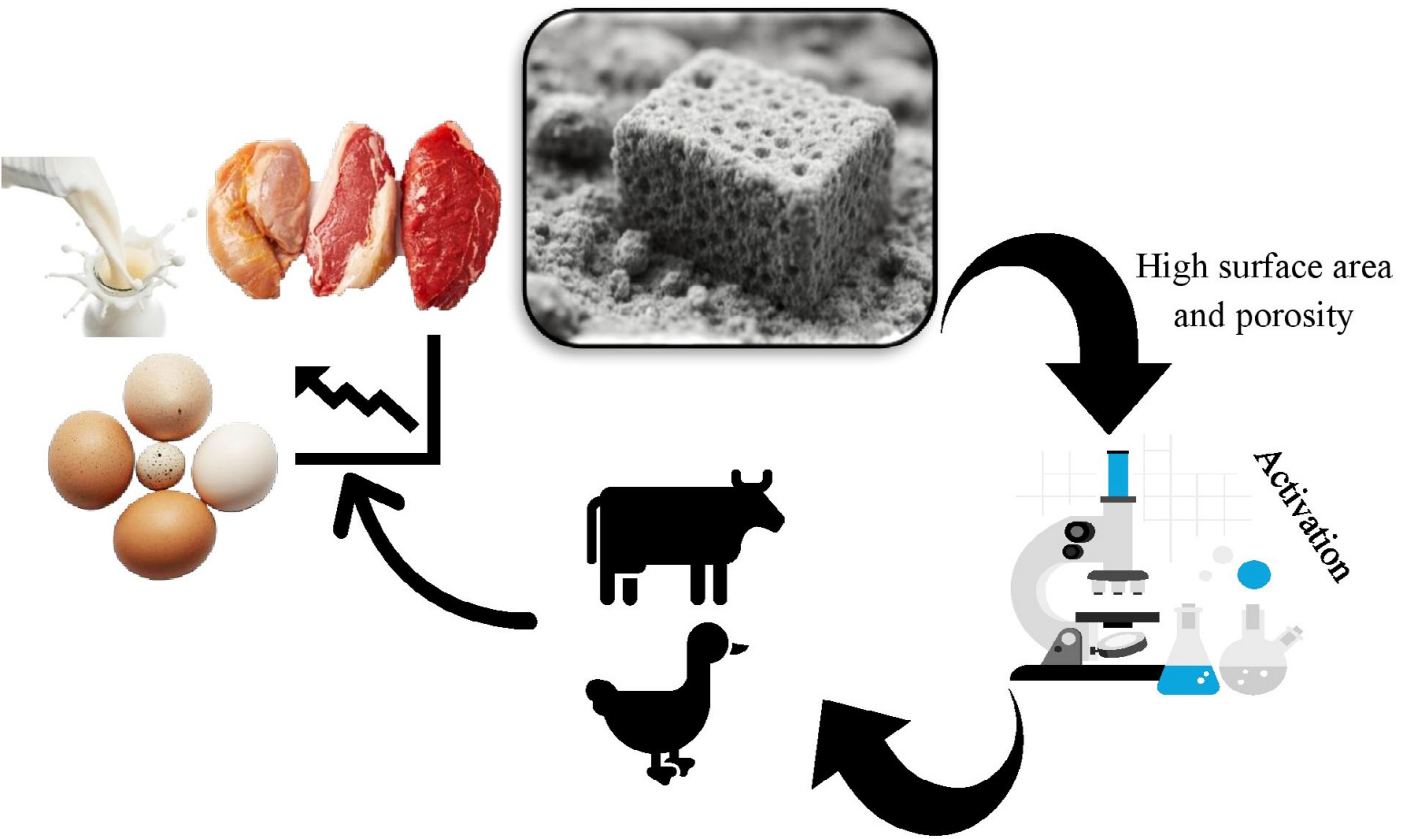
Impact of increasing the surface area and porosity of biochar on livestock performance.

The surface area and porosity of biochar are among its most defining and functionally significant characteristics (Leng et al. 2021), playing a critical role in its effectiveness across various applications, including livestock and poultry production. When an appropriate precursor and optimal pyrolysis conditions are chosen, the surface area and total pore volume of biochar can reach up to 490.8 m^2^/g (Chen et al. 2008). Following successful post‐treatments, such as KOH activation, the surface area and overall pore volume of biochar can be enhanced to as much as 3263 m^2^/g and 1.772 cm^3^/g, respectively (Liu et al. 2016). This extensive surface area results from the complex pore structure formed during pyrolysis, where volatile compounds are driven off, leaving behind a highly porous carbon matrix. The porosity of biochar includes a combination of micro‐, meso‐ and macropores, each contributing to its unique functionality. Micropores (less than 2 nm in diameter) are particularly effective for adsorbing small molecules, such as gases and toxins, whereas mesopores (2–50 nm) and macropores (greater than 50 nm) provide habitat for microorganisms and facilitate the movement of nutrients and water (Leng et al. 2021). The high surface area and porosity of biochar make it an excellent adsorbent, capable of binding a wide range of substances, including toxins, pathogens and nutrients (Osman et al. 2022). The impact of increasing the surface area and porosity of biochar on livestock performance is shown in Figure 2. As shown in this figure, increasing the porosity and surface area of biochar through laboratory techniques can enhance its efficacy as an additive in livestock feed. This improvement, in turn, leads to increased performance and productivity in livestock.

In livestock and poultry production, these properties are particularly beneficial for improving gut health and feed efficiency. For example, biochar can adsorb harmful substances such as mycotoxins, ammonia and heavy metals in the digestive tract, reducing their bioavailability and mitigating their negative effects on animal health (Nair et al. 2023). The detrimental *Campylobacter jejuni* was diminished in the gut microbiome of young hens due to the porous nature of biochar following its administration (Prasai et al. 2016). The porosity of biochar also contributes to its role in manure management. When used as a biological cover on liquid manure storage facilities, biochar can provide various environmental advantages, including diminished odours and gas emissions, as well as improved nutrient retention (Dougherty et al. 2017). Furthermore, the porous structure of biochar enhances its ability to retain nutrients, such as nitrogen and phosphorus, in manure, preventing nutrient leaching and runoff. This not only reduces environmental pollution but also improves the quality of manure as a soil amendment, promoting sustainable agricultural practices. The surface area and porosity of biochar are influenced by several factors, including feedstock type, pyrolysis temperature and heating rate (Ippolito et al. 2020; He et al. 2024). Higher pyrolysis temperatures generally result in greater surface area and porosity due to the more complete decomposition of organic matter and the formation of a more rigid carbon structure. However, excessively high temperatures can also lead to the collapse of pores, reducing biochar's effectiveness. Therefore, optimizing pyrolysis conditions is essential for producing biochar with the desired properties for specific applications (Leng et al. 2021). In summary, the surface area and porosity of biochar are key factors that underpin its multifunctional properties in livestock and poultry production. These characteristics enable biochar to adsorb harmful substances, support beneficial microorganisms and improve nutrient retention, making it a valuable tool for enhancing animal health, feed efficiency and environmental sustainability. Understanding the relationship between biochar's physical structure and its functionality is crucial for maximizing its potential in animal farming systems.

### 3.3 General Characteristics of Biochar

Biochar is a carbon‐rich material produced through the thermochemical conversion of biomass under oxygen‐limited conditions, primarily via pyrolysis or HTC (Amalina et al. 2022). This process yields a substance with a complex array of physical and chemical properties that make it valuable for agricultural and environmental applications. The material possesses a highly porous structure with a range of pore sizes that create habitats for soil microorganisms while simultaneously providing surfaces for nutrient and contaminant adsorption (Zhang et al. 2021; Bolan et al. 2023). Key characteristics of biochar and their implications are shown in Table 1. As shown in the table, biochar's properties and applications vary significantly depending on feedstock type and pyrolysis temperature. Key findings demonstrate that higher temperatures (e.g., 900°C for cow manure) enhance adsorption capacity for dyes, whereas chemical modifications like HNO_3_ treatment reduce heavy metals but increase free radicals. Specific biochars, such as corn straw‐derived biochar (pyrolysed at 800°C), enhance methane production, whereas spirulina‐based biochar (produced at 600°C) effectively removes heavy metals. The data highlight biochar's tunable nature for diverse environmental applications, including wastewater treatment, soil remediation and energy production (Xiang et al. 2020; Kataya et al. 2024). One of biochar's most notable characteristics is its high surface area, which can exceed 3263 m^2^/g after chemical activation (Liu et al. 2016). This extensive surface area, combined with various reactive functional groups on its surface, enables biochar to participate in numerous chemical and biological processes within soil systems. Biochar also possesses certain redox‐active organic functional groups on its surface, such as quinone and phenazine, and can act as an electron mediator or shuttle similar to anthraquinone‐2,6‐disulfonate, which accepts electrons from one microorganism and transfers them to another microorganism (Wang et al. 2021). In this regard, a study aimed at investigating the role of biochar's redox‐active components in denitrification and N_2_O reduction was conducted (Chen et al. 2018). Biochars pyrolysed at 300°C and 800°C were separated into dissolved and condensed aromatic structures to assess their impact on soil denitrifying bacteria. The 300°C biochar enhanced nitrate reduction by acting as an electron donor, whereas the 800°C biochar reduced total denitrified nitrogen by serving as an electron acceptor. Both biochars significantly improved the final denitrification step, reducing N_2_O emissions by 74.1%–99.9%. The 300°C biochar increased nitrate‐reducing bacteria, promoting N_2_O reduction, whereas the 800°C biochar acted as an electron sink and shuttle, further minimizing N_2_O production (Chen et al. 2018). Biochar demonstrates multiple mechanisms for contaminant immobilization, including surface complexation through oxygen‐containing functional groups, interactions with aromatic structures and co‐precipitation with mineral phases (Zhang et al. 2021; Chang et al. 2024). Furthermore, recent research has revealed that biochar can store and gradually release electrons, creating what has been termed an electron battery effect that influences microbial nitrogen‐cycling processes (Prévoteau et al. 2016). These diverse properties elevate biochar beyond a simple soil amendment, positioning it as a multifunctional material capable of modifying soil ecosystems at multiple levels. Its applications extend to carbon sequestration, water retention improvement in arid regions and enhancement of overall soil fertility through its physical and electrochemical interactions with soil components and microorganisms. The material's versatility stems from the interplay between its structural characteristics and surface chemistry, which can be tailored through selection of feedstock and production parameters to meet specific agricultural and environmental needs.

**TABLE 1:** Key characteristics of biochar and their implications.

Feedstock and materials	Characteristic	Description	Implications/Applications	References
**Feedstock**: Cow manure **Pyrolysis temperatures**: 300°C, 500°C, 700°C and 900°C	Functional groups analysed by FTIR/XPS Adsorption capacities for MB and MO Reusability over 10 cycles	CMBC900 showed highest adsorption (200 mg/g MB, 147 mg/g MO) In mixed dyes: 104.5 mg/g MB, 98.7 mg/g MO Excellent regeneration: 97.5% MB, 90.5% MO retention after 10 cycles	Effective conversion of agricultural waste to adsorbent Sustainable wastewater treatment solution High‐temperature biochar (900°C) optimal for dye removal Cost‐effective and reusable technology	Nguyen et al. (2024)
**Feedstocks**: Willow, rice husk, sewage sludge, oilseed rape straw and wheat straw **Modifiers**: HNO_3_ (acid), KOH (alkali) and H_2_O_2_ (oxidizer)	Morphology Elemental composition Crystalline structure HMs, PAHs and EPFRs content	**HNO_3_**: ↓HMs and C_tot_ PAHs but ↑C_free_ PAHs and EPFRs **H_2_O_2_**: Moderate ↓PAHs **KOH**: ↑C_tot_ PAHs (porosity/DOC dependent)	**HNO_3_‐modified**: Optimal for HMs remediation (requires EPFRs management) **H_2_O_2_‐modified**: Balanced PAHs reduction **KOH‐modified**: Feedstock‐specific applications	Hawryluk‐Sidoruk et al. (2024)
**Feedstocks**: Corn straw, *Dicranopteris dichotoma*, bamboo, kitchen waste, tea residues, mushroom waste, cassava lees, chlorella and sargassum **Pyrolysis temperatures**: 300°C, 500°C and 800°C	**Physicochemical properties**: Specific surface area, total pore volume and organic functional groups **Microbial effects**: Enriched functional consortia, modified methanogenic pathways	CS‐800°C, MW‐300°C and S‐500°C biochars significantly improved both Rm and yield Other biochars enhanced either Rm or yield Performance variation depended on feedstock type and pyrolysis temperature	Provides selection criteria for biochar in AD systems CS/MW/S‐derived biochars most effective for methane enhancement Enables optimized waste‐to‐energy conversion Guides feedstock‐temperature combinations for specific AD goals	Wu et al. (2024)
**Feedstocks**: Straw rice, sawdust, sugar cane and tree leaves **Pyrolysis temperatures**: 400°C, 600°C and 800°C	Moisture (1.11%–4.18%), WHC (12.9–27.6 g/g), pH (to 10.4) and nutrients (P to 134.6 mg/kg)	↓Yield with ↑temperature; 800°C enhances chemical properties	Leaves@800°C: pH amendment Sugarcane@800°C: P/Ca source	Khater et al. (2024)
**Feedstocks**: Rice straw and corncob **Pyrolysis temperatures**: 400°C and 600°C	↑Microbial richness Enriches *Caproiciproducens* (chain elongator) Optimizing the metabolic pathways	CC‐400°C: Highest yield RS biochar: Lower efficiency	**For maximum yield**: Use CC biochar at 400°C **Process enhancement**: Biochar boosts key microbes **Waste valorization**: Efficient food waste‐to‐chemical conversion	Tang et al. (2024)
**Feedstock**: Textile sludge **Pyrolysis temperatures**: 300°C, 500°C and 700°C	PAHs reduced 85%–95% 300°C: High Zn/Ni bioavailability 700°C: Low HMs leaching Risk index <150 (safe above 500°C)	TSB‐300°C/700°C: ↑Seed germination TSB‐500: 20%–30% germination inhibition	**For disposal**: Use 700°C (safest for HM immobilization) **For soil use**: 300°C is good (but monitor Zn/Ni) Avoid 500°C	Yadav et al. (2024)
**Feedstock**: B1 and B2 **Pyrolysis temperatures**: 550°C	Water retention capacity Air permeability Ks Pore size distribution	3% biochar: Optimal for water content 6% biochar: Altered air permeability B1 showed greater improvements in mesopores and soil structure Significant Ks improvement in amended soils	**For water retention**: Use 3% biochar (both soil types) **For soil structure**: Prefer twig biochar (i.e., B1) Apply 6% for pore modification **Texture‐specific**: B1 better for sandy soils B2 better for silt soils	Faloye et al. (2024)
**Feedstock**: *Spirulina platensis* **Pyrolysis temperatures**: 400°C and 600°C	N‐doped biochar (pyridine‐N, pyrrole‐N) Functional groups: –COOH, –OH	SP600 > SP400 for Pb/Zn adsorption Mechanisms: Electrostatic, cation exchange and complexation	**Water treatment**: Use SP600 for heavy metal removal **Soil remediation**: Effective for Pb/Zn immobilization **Advantage**: Low‐cost N‐rich feedstock	Myung et al. (2024)

Abbreviations: AD, anaerobic digestion; B1, mango twig; B2, mango branch; Ca, calcium; CC, corncob; CC‐400, corncob pyrolysed in 400°C; C_free_ PAHs, freely dissolved polycyclic aromatic hydrocarbons; CMBC900, biochar produced at 900°C; CS, corn straw; CS‐800, corn straw pyrolysed at 800°C; C_tot_ PAHs, solvent extractable polycyclic aromatic hydrocarbons; EPFRs, environmentally persistent free radicals; FTIR, Fourier transform infrared spectroscopy; HMs, heavy metals; Ks, saturated hydraulic conductivity; MB, methylene blue; MO, methyl orange; MW, mushroom cultivation waste; MW‐300°C, mushroom waste pyrolysed at 300°C; N, nitrogen; Ni, nickel; P, phosphorus; PAHs, polycyclic aromatic hydrocarbons; Pb, lead; Rm, methane production rate; RS, rice straw; S, sargassum; SP, *Spirulina platensis*; SP400 and SP600, *Spirulina platensis* pyrolysed at 400°C and 600°C, respectively; TSB, textile sludge; TSB‐300°C/700°C, textile sludge pyrolysed at 300°C and 700°C, respectively; WHC, water‐holding capacity; XPS, x‐ray photoelectron spectroscopy; Zn, zinc.

### 3.4 Adsorption Capacity of Different Types of Biochar

The adsorption capacity of biochar is a critical factor that determines its effectiveness in various environmental applications, particularly in soil enhancement and pollution remediation. Biochar, produced through the pyrolysis of organic materials, exhibits unique physical and chemical properties that influence its ability to adsorb nutrients and contaminants. Different types of biochar, derived from various feedstocks and produced under different conditions, possess distinct functional groups and surface characteristics that directly impact their adsorption capabilities (Dong et al. 2023; He et al. 2024). First, the surface area and porosity of biochar play significant roles in its adsorption capacity. Biochars with higher surface areas and greater porosity typically provide more active sites for adsorption, allowing for increased interaction with both nutrients and pollutants (Tomczyk et al. 2020; Bolan et al. 2023). Adsorption capacity of different types of biochar is shown in Table 2. As shown in the table, biochar's adsorption capacity depends on feedstock and pyrolysis temperature. Higher temperatures (e.g., 600–800°C) reduce oxygen content but enhance carbon stability and micropore formation, whereas lower temperatures (300–500°C) retain functional groups (–OH, C = O) beneficial for soil health and metal adsorption. Manure‐based biochars show higher phosphorus sorption, whereas modified biochars (e.g., KOH‐treated water hyacinth) improve NH_4_⁺–N adsorption. Overall, optimal performance balances pore structure, functional groups and pyrolysis conditions for specific applications. Research conducted by Ippolito et al. (2020) reveals that wood‐derived biochar contains a higher percentage of carbon (70.5%) compared to biochar made from crop waste (61.4%), grass (63.6%) and manure or biosolids (41.6%). The increased carbon content plays a key role in strengthening concrete when biochar is used as a filler. There are notable differences between biochar from hardwoods and softwoods. Hardwood biochar tends to have a more even pore distribution, whereas softwood biochar generally provides a larger surface area. Wood‐based biochar also demonstrated the highest surface area (184 m^2^/g) among all feedstock types, which helps improve bonding with cement paste in concrete mixtures. On the other hand, biochar from herbaceous materials like crop residues and grasses often has higher ash content (21.1% and 18.0%, respectively) and more mineral content than wood‐based biochar (10.2%). These properties can influence the workability and final strength of concrete in practical applications (Zhou et al. 2025). Furthermore, the presence of functional groups such as carboxyl, hydroxyl and phenolic groups on the surface of biochar further enhances its ability to bind with various ions and molecules (Bolan et al. 2023). These functional groups can form electrostatic attractions and hydrogen bonds with positively charged nutrients like ammonium (NH_4_⁺) and potassium (K^+^), making certain biochars particularly effective in nutrient retention (Hossain et al. 2020; Kohira et al. 2024). The feedstock used in biochar production significantly influences its adsorption properties. Biochar serves as an important source of plant nutrients, especially when produced from manure and organic residues at lower pyrolysis temperatures (≤400°C) (Hossain et al. 2020). Unlike biochar derived from crop residues or woody biomass, manure‐based biochar typically contains higher concentrations of nitrogen, phosphorus, potassium and other micronutrients essential for plant growth (Hossain et al. 2020). Multiple studies have shown that biochar generated from biosolids and animal waste generally exhibits richer nutrient profiles compared to plant‐based feedstocks like wood or straw (Liu et al. 2020; Heinrich et al. 2023). This enhances the bioavailability of key elements such as phosphorus and nitrogen by facilitating their release from microbial biomass. Furthermore, applying such nutrient‐rich biochar can help minimize nitrate leaching in agricultural soils, improving nutrient retention. This characteristic makes manure‐based biochar an excellent candidate for improving soil fertility while minimizing nitrogen leaching into groundwater. The pyrolysis temperature during the production of biochar is another crucial factor affecting its adsorption capacity. Higher pyrolysis temperatures generally lead to increased carbonization, resulting in biochar with a more stable structure and higher surface area (Ippolito et al. 2020). However, excessively high temperatures can also reduce the number of functional groups available for adsorption. Thus, an optimal pyrolysis temperature must be identified to balance stability and adsorption capacity. Studies have shown that biochars produced at moderate temperatures (around 500–600°C) often exhibit the best performance in terms of nutrient and contaminant adsorption (Bolan et al. 2023). In addition to the physical and chemical properties of biochar itself, the environmental conditions in which it is applied also play a significant role in determining its effectiveness. Soil pH, moisture content and the presence of competing ions can all influence the adsorption dynamics of biochar (Zhu et al. 2025). For instance, in acidic soils, biochar can enhance the availability of nutrients by altering the pH and providing a more favourable environment for nutrient adsorption (Gao et al. 2025). Conversely, in alkaline conditions, the competition between various ions can hinder the adsorption capacity of biochar (Yan et al. 2025). Furthermore, the interaction between biochar and soil microorganisms is an essential aspect of its functionality. Biochar can enhance microbial activity, which, in turn, can influence the bioavailability of nutrients and the degradation of pollutants (Bolan et al. 2023). The adsorption capacity of biochar can be further enhanced by its ability to provide a habitat for beneficial microorganisms, creating a synergistic effect that improves soil health and productivity. Furthermore, it has been reported that when the levels of metal ions like potassium, calcium, sodium and magnesium on biochar surfaces become excessively elevated, they tend to compete with heavy metal ions. This competition diminishes the effectiveness of ion exchange and obstructs the heavy metal adsorption process (Alothman et al. 2015; Wang et al. 2022). Therefore, the efficiency of ion exchange is influenced by the concentration of these metal ions present on the biochar surface. In a recent study analysing 465 papers, it has been shown that biochar's effectiveness in reducing soil emissions and nutrient loss depends on feedstock type and pyrolysis temperature. Wood‐based biochar exhibits higher porosity, whereas straw‐based biochar contains more O, N and H, with low‐temperature production enhancing adsorption through functional groups. Molecular simulations further reveal how these properties influence biochar–soil interactions, guiding optimal selection for agricultural applications (He et al. 2024). In summary, the adsorption capacity of different types of biochar is influenced by various factors, including feedstock type, production conditions and environmental context. Understanding these factors is vital for optimizing biochar applications in agriculture and environmental management. By selecting the appropriate type of biochar based on its adsorption characteristics, practitioners can enhance soil fertility, reduce nutrient leaching and mitigate pollution effectively.

**TABLE 2:** Adsorption capacity of different types of biochar.

Feedstock	Pyrolysis temperature (°C)	Key functional groups	Key finding	Reference
Douglas fir wood	623–873	C = O, –COOH and –OH	Higher pyrolysis temperature reduces oxygen and volatile content, lowering O/C and H/C ratios Micropores (<1 nm) form but are only detectable via CO_2_ adsorption, not N_2_ Surface charge and reactivity drop as oxygen‐containing groups break down at higher temperatures	Suliman et al. (2016)
*Phragmites australis* straw and *Spartina alterniflora* straw	300–500	–OH, C = O, –CH and –C–O–C	Higher temperature biochars, especially from *S. alterniflora* at 500°C, have greater potential for carbon sequestration Lower temperature biochars, with higher DOC, are beneficial for soil health and microbial activity	Wang et al. (2021)
Water hyacinth, chicken manure and wood	300°C and 600°C	O–H, C–H, C = C, C–H, C–O and C–O–C	10% WHB at 300°C showed highest water retention CMB performed better than WB at 10% amendment All biochars at 10% amendment improved water retention in both loose and dense soils Wetting/Drying capacity remained constant at corresponding RH levels Effects were observed at both 300°C and 600°C pyrolysis temperatures	Huang et al. (2021)
Wood, rice husk and sunflower husk	100–700°C	–OH, phenolic and esters	**Adsorption efficiency**: Wood > rice husk > sunflower husk **Pore characteristics**: Rice husk had largest total pore volume, and wood had most thermally stable and irregular pores **Performance**: All biochars achieved >77% metal removal, enhancing Cu/Pb adsorption by 9%–19% and Zn by 11%–21%	Burachevskaya et al. (2023)
Wood shavings	450°C	–OH, –COOH, C = O and –CN	Biochar exhibits enhanced adsorption capacity for NO_2_ due to functional groups	Zbair et al. (2024)
Water hyacinth	350°C	–OH and –COOH	KOH and H_2_O_2_ modifications significantly enhanced NH_4_^+^–N adsorption capacity of biochar	Kohira et al. (2024)
*Parthenium* weed, corn cobs, farmyard manure and poultry manure	400°C and 600°C	–OH and –COOH	Manure‐based biochars showed higher phosphorus sorption at 400°C, whereas plant‐based biochars had lower sorption capacities	Musa et al. (2024)
Grub manure	450°C, 600°C, 750°C	Aromatic structures and –OH	Higher pyrolysis temperatures enhance the stability and surface pore structure of grub manure‐derived biochar. Toxic elements in grub manure are reduced after pyrolysis	Zhao et al. (2024)

Abbreviations: C, carbon; CMB, chicken manure; H, hydrogen; O, oxygen; WB, wood biochar; WHB, water hyacinth.

### 3.5 Nutrient Content and Adsorption Capacity

**FIGURE 3:**
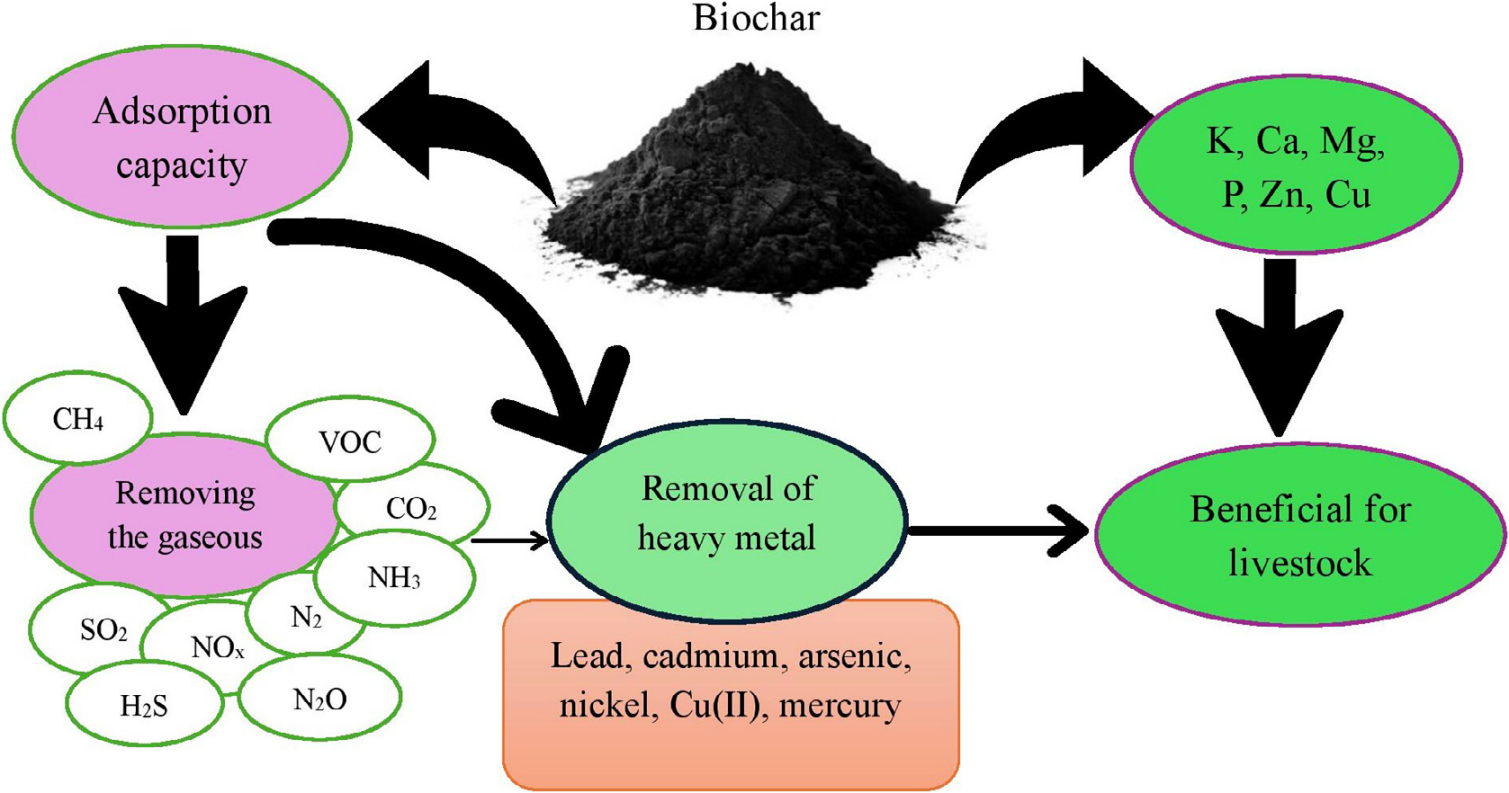
Some nutrient content and adsorption capacity of biochar. VOC, volatile organic compound.

Biochar is not only a carbon‐rich material but also a valuable source of essential nutrients that can enhance the nutritional profile of animal diets and improve soil fertility when used in manure management. The nutrient content of biochar varies depending on the feedstock and pyrolysis conditions, with common nutrients including various minerals. For instance, biochar obtained from animal waste, poultry waste, seaweed and agricultural residues contains higher nutrient concentrations, exhibits a higher pH and has less stable carbon compared to biochar made from woody biomass (Kavitha et al. 2018; Alkharabsheh et al. 2021). When incorporated into animal feed, biochar can supplement dietary minerals, supporting metabolic processes, bone development and overall animal health. This is particularly beneficial in regions where feed quality is suboptimal or where mineral deficiencies are prevalent. In addition to its nutrient content, biochar's adsorption capacity is a key feature that contributes to its effectiveness in livestock production (Farooq et al. 2023). The high surface area and porous structure of biochar enable it to adsorb a wide range of substances, including nutrients, toxins and gases (Wen et al. 2023). This adsorption capacity plays a dual role: It enhances nutrient retention and availability while reducing the bioavailability of harmful compounds. For example, biochar can retain nitrogenous compounds in the digestive tract, preventing their loss through excretion and improving nitrogen utilization efficiency in poultry (Prasai et al. 2018). This not only enhances feed efficiency but also reduces nitrogen emissions, contributing to environmental sustainability. Biochar's adsorption capacity also extends to its role in manure management. When added to animal manure, biochar can adsorb nutrients such as nitrogen and phosphorus, preventing their loss through leaching or volatilization (Chen et al. 2021). This nutrient retention capability improves the quality of manure as a soil amendment, promoting sustainable agricultural practices. Furthermore, biochar's ability to adsorb odorous compounds, such as ammonia and hydrogen sulphide, helps reduce emissions and improve air quality in livestock facilities, creating a healthier environment for both animals and workers (Song et al. 2021; Sobol and Dyjakon 2022). The adsorption capacity of biochar is influenced by its physicochemical properties, including surface area, porosity and the presence of functional groups such as carboxyl, hydroxyl and phenolic groups (Nosratabad et al. 2024; Eissa et al. 2024). These functional groups enable biochar to participate in ion exchange and chelation reactions, further enhancing its ability to adsorb nutrients and toxins. The nutrient content and adsorption capacity of biochar are shown in Figure 3. As illustrated in the figure, certain minerals, such as calcium, phosphorus, potassium, magnesium, zinc and copper, are present in various types of biochar, and their consumption by livestock can provide nutritional benefits. Additionally, different types of biochar possess the capacity to adsorb various gases (such as methane, carbon dioxide, NH_3_, N_2_ and SO_2_) and heavy metals (including lead, cadmium and mercury). The adsorption of these substances can, through various mechanisms, have positive effects on livestock. In summary, the nutrient content and adsorption capacity of biochar make it a versatile and multifunctional material for livestock and poultry production. By supplementing essential nutrients, enhancing nutrient retention and reducing the bioavailability of harmful substances, biochar contributes to improved animal health, feed efficiency and environmental sustainability. Understanding the interplay between biochar's nutrient content and adsorption properties is crucial for optimizing its use in animal farming systems and maximizing its benefits.

### 3.6 Stability and Degradation in Animal Systems

The stability of biochar in animal systems is a critical factor that determines its long‐term effectiveness and safety as a feed additive or manure amendment. During the pyrolysis process, biomass undergoes thermal decomposition, resulting in the formation of organic carbon characterized by condensed aromatic configurations. These structures exhibit remarkable resistance to degradation, as demonstrated by the persistence of biochar in soil environments for extended periods ranging from centuries to millennia (Lehmann 2009; Nguyen et al. 2010). Furthermore, biochar is known for its high degree of recalcitrance, meaning it is resistant to microbial and chemical degradation. This stability is primarily due to its carbon‐rich composition and the formation of aromatic carbon structures during pyrolysis, which are difficult for microorganisms to break down (Lehmann 2009; Nguyen et al. 2010). For example, the addition of biochar mixed with molasses in cow feed has demonstrated the potential to reduce farming costs, enhance soil properties and improve pasture health, with dung beetles facilitating the incorporation of nutrient‐rich biochar into the soil. As a result, biochar can persist in the digestive tract of animals and in manure for extended periods, providing sustained benefits such as toxin adsorption, nutrient retention and gut health modulation. In the digestive systems of livestock and poultry, biochar's stability ensures that it remains intact as it passes through the gastrointestinal tract, allowing it to continuously adsorb harmful substances such as mycotoxins, pathogens and gases. This prolonged activity helps maintain a healthier gut environment, reducing the risk of digestive disorders and improving nutrient absorption. However, the stability of biochar does not mean it is entirely inert. For instance, research has shown that biochar derived from poultry litter, as an additive, can mitigate the adverse effects of aflatoxin B1 on the performance, haematological parameters and immune responses of broiler chickens (Rashidi et al. 2020).

Over time, biochar may undergo slow oxidation and physical breakdown, particularly in the acidic gastric environment or through enzymatic action. This gradual degradation could release adsorbed nutrients and minerals, potentially providing supplemental benefits to the host animal. Supporting this, Leng (2014) demonstrated that biochar enhances rumen microbial populations by offering favourable surface areas for colonization, thereby increasing ATP production and improving feed digestibility. Further evidence suggests that biochar supplementation boosts animal production performance, primarily by modulating gastrointestinal microbiota and enhancing nutrient digestibility (Han et al. 2018). The study demonstrates that adding manure‐based biochar increases soil moisture retention, contributing to sustainable agriculture practices (Rehman et al. 2020). The slow degradation of biochar in manure ensures that it continues to adsorb ammonia, hydrogen sulphide and other odorous compounds, improving air quality and reducing environmental pollution (Hammerschmiedt et al. 2022). Additionally, the persistence of biochar in manure enhances its value as a soil amendment, as it can improve soil structure, water retention and nutrient availability for extended periods after application (Hammerschmiedt et al. 2022). Despite its stability, the degradation of biochar in animal systems is influenced by several factors, including particle size, pyrolysis temperature and the presence of microorganisms. Smaller biochar particles may degrade more quickly due to increased surface area exposure, whereas biochar produced at higher pyrolysis temperatures tends to be more stable due to the formation of more rigid carbon structures (Tomczyk et al. 2020). Microbial activity in the gut or manure can also contribute to the gradual breakdown of biochar, releasing adsorbed nutrients and minerals into the system. In general, the stability and slow degradation of biochar in animal systems are key attributes that enhance its effectiveness as a feed additive and manure amendment. By providing sustained benefits such as toxin adsorption, nutrient retention and environmental protection, biochar contributes to improved animal health, productivity and sustainability. Understanding the factors that influence biochar's stability and degradation is essential for optimizing its use in livestock and poultry production systems.

## 4 Effect of Biochar on Livestock

### 4.1 Impact on Gut Health and Microbiota

**FIGURE 4:**
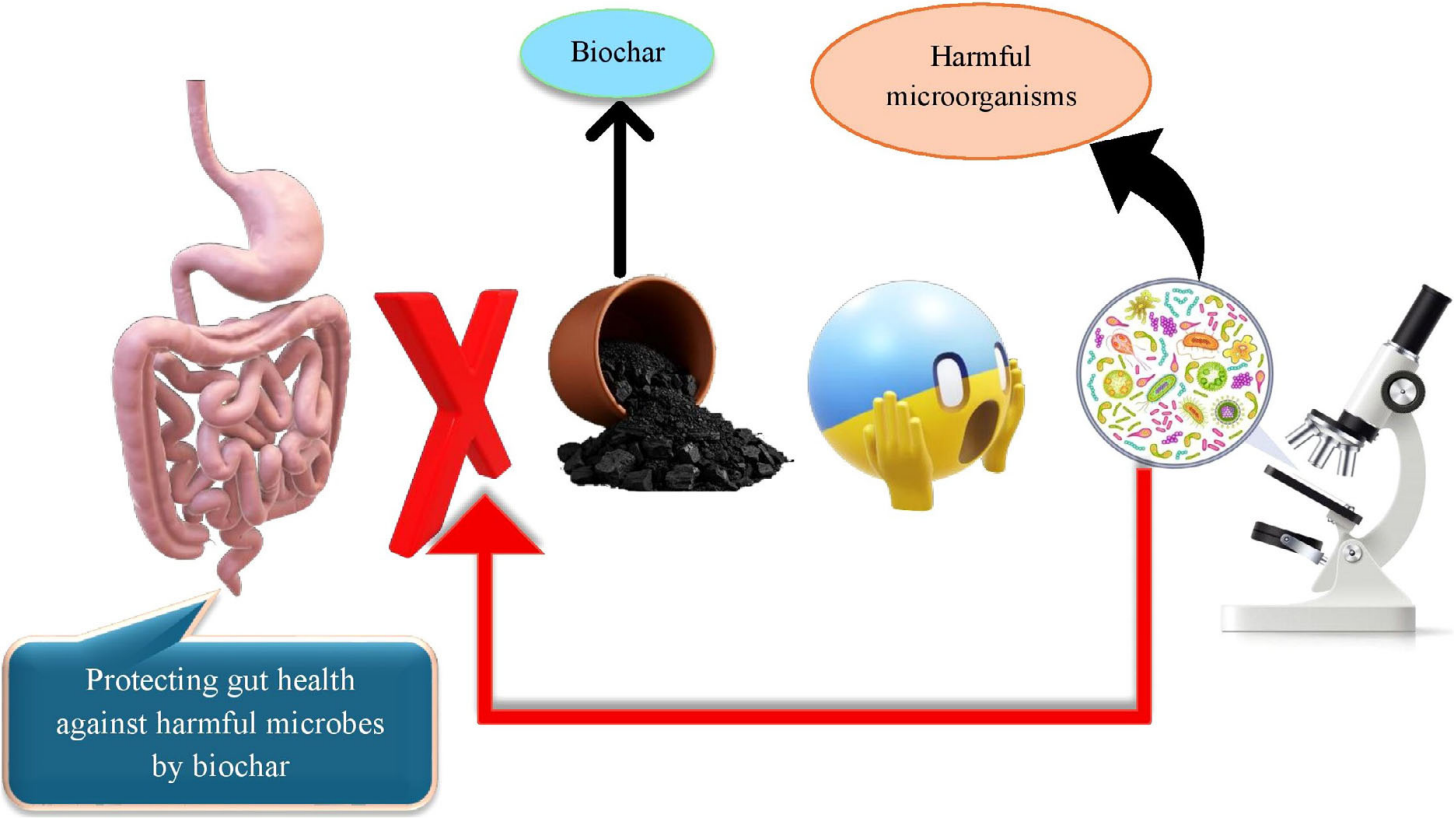
Effects of biochar on small intestine health.

The gut health of livestock and poultry is a cornerstone of their overall well‐being, influencing growth performance, disease resistance and nutrient utilization (Naeem and Bourassa 2025). Biochar has emerged as a promising tool for enhancing gut health and modulating the gut microbiota, thanks to its unique physicochemical properties. Gut microbiota refers to the diverse community of trillions of microorganisms (bacteria, fungi, viruses and others) living in an animal's digestive tract. These microbes play vital roles in digestion, immune function and overall health by breaking down feed components, producing nutrients like vitamins and protecting against pathogens. For instance, the incorporation of humid litter biochar and probiotics improved performance and morphological characteristics of the small intestine in broiler chickens subjected to cold stress (Hasanvand et al. 2023). Moreover, biochar can adsorb harmful substances, including toxins, heavy metals and mycotoxins within the gastrointestinal system, thereby reducing their bioavailability and improving animal health (Burezq and Khalil 2025). The porous structure and high surface area of biochar enable it to bind toxins, pathogens and gases, reducing their negative impact on gut health. For instance, biochar can adsorb mycotoxins, which are toxic compounds produced by fungi that often contaminate feed and disrupt gut integrity (Ahmadou et al. 2021; Appell et al. 2023). By neutralizing these toxins, biochar helps maintain the structural and functional integrity of the gut lining, preventing issues such as leaky gut syndrome and inflammation. In addition to its adsorption capabilities, biochar plays a significant role in shaping the gut microbiota, the complex community of microorganisms that reside in the digestive tract. A balanced gut microbiota is essential for efficient digestion, immune function and overall health. For example, adding 2% (w/w) biochar to laying diets significantly reduced pathogenic bacteria and positively altered the intestinal microbiota, demonstrating its potential as an effective alternative to antibiotics in the poultry industry (Willson et al. 2019). Furthermore, biochar enhances nutrient digestibility and promotes microbial proliferation, adsorbs harmful substances, minimizes energy wastage and fosters an optimal environment for advantageous microorganisms, ultimately leading to enhanced performance in animals (Sirjani et al. 2023). However, hardwood biochar appears not to affect the ruminal fermentation characteristics but may reduce enteric methane at high inclusion levels by altering ruminal microbial populations (Teoh et al. 2019). Biochar (an organic medicinal charcoal) has been shown to promote the growth of beneficial bacteria, such as *Lactobacillus*, while inhibiting the proliferation of pathogenic bacteria, such as *Escherichia coli*, in manure (Kim et al. 2017). This shift in microbial balance is attributed to biochar's ability to create a favourable environment for beneficial microbes by adsorbing harmful substances and providing a porous habitat for microbial colonization. Initial findings indicate that chestnut biochar, particularly at low concentrations, exhibits selective prebiotic effects by stimulating *Lactiplantibacillus plantarum* growth while suppressing pathogenic *E. coli* strains (Reggi et al. 2025). Another important mechanism by which biochar impacts gut health is through its ability to modulate pH levels in the digestive tract. By adsorbing excess acids or bases, biochar helps maintain a stable pH, which is crucial for optimal enzyme activity and nutrient absorption (Wang et al. 2017; Viaene et al. 2024). This pH stabilization also creates an environment that is less conducive to the growth of pathogenic bacteria, further supporting gut health. Additionally, biochar's mineral content, including calcium, magnesium and potassium, can contribute to the buffering capacity of the gut, enhancing its resilience to dietary changes or stressors. The impact of biochar on gut health extends beyond the digestive tract, influencing systemic health and productivity. A healthy gut microbiota supported by biochar can enhance immune function, reducing the incidence of infections and the need for antibiotics (Panwar et al. 2021; Chu et al. 2023). This not only improves animal welfare but also aligns with the growing demand for antibiotic‐free animal production. Moreover, by improving nutrient absorption and reducing the energy expenditure associated with combating toxins and pathogens, biochar can enhance feed efficiency and growth performance, leading to better economic outcomes for farmers. In summary, biochar's impact on gut health and microbiota is multifaceted, involving adsorption of harmful substances, modulation of microbial balance, stabilization of pH and provision of essential minerals. These mechanisms collectively contribute to improved gut integrity, enhanced nutrient utilization and overall animal health. By supporting a balanced and resilient gut ecosystem, biochar offers a sustainable and effective solution for optimizing livestock and poultry production systems. Effects of biochar on small intestine health are shown in Figure 4. As evidenced by the figure, harmful microbes exist that, if they enter the digestive system, can cause irreparable damage to the gastrointestinal tract of livestock, particularly their intestines. Biochar, with its porous structure and specific surface area, is capable of adsorbing these microbes, thereby neutralizing or diminishing their effects within the digestive system. Consequently, biochar can play a significant role in maintaining intestinal health against pathogenic agents.

### 4.2 Modulation of Immune Responses

The immune system of livestock and poultry is a critical component of their overall health, influencing their ability to resist infections, respond to vaccines and maintain productivity (Vlasova and Saif 2021). To evaluate the immediate impact of biochar on immune cells, researchers used mammalian macrophages and found that biochar significantly reduced pro‐inflammatory cytokine levels in activated macrophages (Yıldızlı et al. 2021). One of the primary ways biochar influences immune function is through its impact on gut health. A healthy gut is closely linked to a robust immune system, as a significant portion of immune cells resides in the gut‐associated lymphoid tissue. By adsorbing harmful substances such as toxins, pathogens and allergens, biochar helps maintain gut integrity and reduces the burden on the immune system. This allows the immune system to function more efficiently, focusing on combating genuine threats rather than being overactivated by constant exposure to irritants. Biochar's porous structure and high surface area also provide a habitat for beneficial microorganisms, which play a key role in immune modulation. Studies show biochar enhances production of acetic and caproic acids in broilers ceca (Goiri et al. 2021). Additionally, biochar's ability to adsorb harmful bacteria and toxins reduces the risk of systemic infections, further supporting immune function (Li et al. 2024). Another mechanism by which biochar modulates immune responses is through its mineral content. Biochar derived from certain feedstocks contains essential minerals such as zinc, selenium and magnesium, which are known to play vital roles in immune function. For example, zinc is crucial for the development and function of immune cells (Shankar and Prasad 1998), whereas selenium acts as an antioxidant, protecting cells from oxidative stress (Zhang et al. 2023). By supplementing these minerals, biochar can enhance the immune system's ability to respond to infections and recover from illnesses. Biochar's impact on immune responses also extends to its role in reducing stress and inflammation. Chronic stress and inflammation can suppress immune function, making animals more susceptible to diseases (Martin et al. 2011). Biochar's ability to adsorb harmful gases, such as ammonia, in the gut and environment helps reduce stress levels in animals, creating conditions that support optimal immune function. Furthermore, biochar's anti‐inflammatory properties, mediated through its interaction with gut microbiota and immune cells, can help mitigate inflammation and promote faster recovery from infections or injuries (Forbes et al. 2016; Yoo et al. 2020). In summary, biochar's modulation of immune responses is a multifaceted process that involves maintaining gut health, supporting beneficial microbiota, providing essential minerals and reducing stress and inflammation. By enhancing immune function, biochar contributes to improved disease resistance, reduced reliance on antibiotics and overall better health and productivity in livestock and poultry. This makes biochar a valuable tool for promoting sustainable and resilient animal production systems.

### 4.3 Effects on Growth Performance

Nutritional effects of biochar in different animals are shown in Table 3. As shown in the table, biochar improves growth performance and digestibility in sheep/goats (1%–2% diet) and turkey poults (5–25 g/kg starter) but shows neutral effects in dairy cows. Benefits are dose‐dependent. The use of biochar as a feed additive in livestock production has gained significant attention due to its potential to enhance animal health, improve feed efficiency and reduce environmental impacts (Nair et al. 2023). When incorporated into animal diets, biochar serves as a multifunctional supplement that addresses several challenges in modern livestock farming. In the last 10 years, the incorporation of biochar as a feed component in livestock production has significantly increased, as it enhances the digestion and metabolic processes of nutrients (Chen et al. 2019). The inclusion of biochar in livestock diets has been shown to positively influence growth performance, making it a valuable tool for enhancing productivity in animal farming systems. Conversely, findings from an investigation indicated that incorporating mineral biochar into the diet of weaned calves did not yield any statistically significant impact on dry matter intake, daily weight increment, cumulative weight gain or average feed conversion efficacy (Saeidi Garaghani et al. 2023). Ewes receiving a diet enriched with biochar demonstrated an enhancement of 8.1% in body mass and a 26% improvement in body condition score (Burezq and Khalil 2025). One of the primary ways biochar contributes to improved growth performance is through its ability to enhance feed efficiency. Biochar's porous structure and high surface area enable it to adsorb nutrients and release them gradually in the digestive tract, ensuring optimal nutrient absorption. This process reduces nutrient losses through excretion and maximizes the utilization of dietary components, leading to better weight gain and feed conversion ratios. For instance, it can be reported that the incorporation of biochar into the diet at concentrations of 1%, 2%, 4% and 6% enhanced the physiological condition and growth performance of broiler chickens, without negatively affecting the haematological parameters of broiler chicks (Elghalid 2022). Another key factor contributing to biochar's positive effects on growth performance is its role in maintaining gut health. Additionally, biochar maintains gut health, which is essential for efficient digestion and nutrient absorption. For example, 1% rice husk biochar in poultry feed significantly reduced plasma triglycerides, coliform counts and *E. coli* levels, though it did not affect weight gain or feed conversion (Hien et al. 2018). In summary, biochar's effects on growth performance are multifaceted, involving improved feed efficiency, enhanced gut health, reduced stress and provision of essential minerals. These benefits collectively contribute to better weight gain, higher feed conversion ratios and overall improved productivity in livestock. As a natural and sustainable feed additive, biochar offers a promising solution for enhancing growth performance and supporting the economic viability of livestock farming systems.

**TABLE 3:** Nutritional effects of biochar in different animals.

Feedstock for biochar preparation	Animal	Suggested amount	Main effects	Reference
Wood chips	Lactating dairy cows	1% of dietary DM	No significant impact of biochar on milk yield, quality or methane emissions No effect on digestive efficiency or animal health No negative effect on animal health and performance	Dittmann et al. (2024)
Pure ash wood	Dairy cows	200 g/cow/day or 1.04% of total daily ration	No significant changes in milk yield, composition or overall methane emissions with biochar supplementation Increase in lignin intake with biochar and increase in crude protein intake with biochar and urea supplementation No effect on dry matter, energy and utilizable protein intake	Terler et al. (2023)
Pine‐source biochar	Grazing beef cow and calf	450 g/animal/day	No significant impact on cow and calf performance or rumen parameters with biochar inclusion Reduction in faecal oocyst counts and increase in carbon: nitrogen ratio observed with biochar; negligible decrease in enteric methane emissions (∼6%)	Damiran et al. (2024)
Corncob	Ram	1.5–4.5 g/day	Enhanced dry matter and crude protein intake with 1.5 g/day biochar Improved daily weight gain and feed conversion ratio in sheep Positive impact on dressing percentage and various carcass weights	Keba et al. (2023)
Lodgepole pine	Sheep	2% of dietary DM	Increased dry matter digestibility and digestible dry matter intake Increased dry matter digestibility and digestible dry matter intake Lambs showed lower preference for biochar ration compared to control, yet incorporated significant amounts into their diets (∼40%) No significant differences in average daily gain or feed conversion efficiency among treatment groups	McAvoy et al. (2020)
Walnut shell and chicken manure	Milking Kermanian ewes and in vitro study	0.5%, 1% and 1.5% dietary DM	1% walnut shell biochar and 1.5% chicken manure biochar reduced methane production and ammonia‐N concentrations while increasing rumen pH in vitro Enhanced milk yield, protein content and solids not fat with biochar inclusion Improved digestibility coefficients for dry matter and organic matter with biochar supplementation Increased blood glucose and total protein levels in ewes fed biochar‐enriched diets	Mirheidari et al. (2019)
Pine‐source biochar	Awassi lambs	1%, 2% and 3% of dietary DM	Enhanced growth performance with 1% biochar Improved feed conversion efficiency observed at 1% biochar level Significant increases in nutrient digestibility metrics with moderate biochar inclusion	Amean and Shujaa (2020)
Pine and coconut	In vitro ruminal fermentation	2.25%–22.5% of dietary DM	Influence on dry matter disappearance in barley silage diets Variability in effects based on biochar source and particle size No significant impact on total gas and methane production	Tamayao et al. (2022)
Corn stover	Broiler birds	2%, 4% and 6% of dietary DM	Enhancement of growth metrics in broiler birds Improvement in haematological parameters Reduction in serum cholesterol and low‐density lipoprotein levels	Dim et al. (2018)
Poultry organic waste materials	Turkey poults	5, 15 and 25 g/kg starter	Improved growth performance with 5 and 15 g/kg biochar supplementation Enhanced haematological parameters with 25 g/kg biochar Reduction in serum biochemical markers (AST, ALT, ALP, urea, creatinine and bilirubin) with 5 and 15 g/kg biochar Increased antioxidant enzyme activity (catalase, glutathione peroxidase) with 15 and 25 g/kg biochar	Dim et al. (2022)
*Miscanthus* grass	Male turkey	5%, 10% and 20% of dietary DM	Enhancement of body weight and body weight gain with 20% biochar in bedding Improved feed conversion ratio with higher feed pellet quality Potential reduction in ammonia production when using biochar as a litter amendment Opportunities for reusing litter combined with biochar and *Miscanthus* grass to improve overall turkey health and performance	Flores et al. (2021)
Commercial biochar (Kuhpayeh, Kerman, Iran)	Laying hens	0%, 25%, 50%, 75% and 100% replacement of biochar with mineral supplements of diet	Low‐cost mineral compound for agricultural use Acts as a fertilizer to enhance plant growth Potential replacement for mineral supplements in poultry diets No negative impact on eggshell quality in laying hens	Ahmadi et al. (2022)
Unknown	Goat	1% of dietary DM	Enhancement of daily live weight gain and feed conversion efficiency Improvement in dry matter digestibility and nitrogen retention Reduction in methane/carbon dioxide ratio in eructed breath when combined with water spinach	Silivong and Preston (2015)
Rice husk	Goat	0.5%, 1.0% and 1.5% of dietary DM	Curvilinear responses in growth criteria: positive effects from 0% to 0.8% biochar, decline at 1.3% Linear decrease in methane production with increased biochar supplementation Enhanced microbial habitat provided by biochar, reducing toxic effects of HCN in cassava diets	Hang et al. (2019)

Abbreviations: ALP, alkaline phosphatase; ALT, alanine aminotransferase; AST, aspartate aminotransferase; HCN, hydrogen cyanide.

### 4.4 Influence on Meat and Milk Quality

The use of biochar in livestock diets not only enhances growth performance but also significantly improves the quality of meat and milk, enhancing the value of animal‐derived products. One primary mechanism by which biochar influences meat quality is through mitigating oxidative stress in animals (Nair et al. 2023). Oxidative stress, caused by an imbalance between free radicals and antioxidants, can compromise meat quality by promoting lipid oxidation, which results in off‐flavours, discolouration and reduced shelf life (Huang and Ahn 2019; Muzolf‐Panek et al. 2024). Notably, replacing 75% of dietary mineral supplements with biochar maintained broiler growth performance while improving meat quality parameters (pH, redness and drip loss) (Kashef et al. 2021). Biochar's antioxidant properties, attributed to its carbon structure and mineral content, help neutralize free radicals, thereby preserving meat freshness and nutritional value. For instance, corncob biochar supplementation significantly increased ovine growth metrics, including dressing yield, carcass weights and ribeye muscle area, while maintaining organ weights within normal ranges (Keba et al. 2023). Similarly, 2% pine‐derived biochar improved lean carcass yield in feedlot cattle (Terry et al. 2020). Beyond antioxidant effects, biochar modifies meat fatty acid profiles (Domaradzki et al. 2022). Studies report increased beneficial fatty acids like omega‐3 species (Islam et al. 2014), enhancing nutritional quality, flavour and texture. Specifically, biochar‐supplemented diets produced meat with improved tenderness and juiciness (Farghly et al. 2023). Biochar's benefits extend to milk quality, where it increases protein, fat and lactose contents (Tahery et al. 2023; Benhissi et al. 2025). These improvements stem from enhanced nutrient absorption and reduced mycotoxin contamination. Crucially, biochar lowers somatic cell counts (SCC) (Schmidt et al. 2019), indicating improved udder health and reduced mastitis incidence (Panchal et al. 2024). By promoting gut health and reducing systemic inflammation, biochar helps improve overall animal health, leading to lower SCC levels and higher quality milk. In summary, biochar's influence on meat and milk quality is multifaceted, involving antioxidant effects, improvements in fatty acid profiles, enhanced nutrient utilization and reductions in harmful substances. These benefits collectively contribute to higher quality animal‐derived products that are more nutritious, flavourful and safe for consumers. By incorporating biochar into livestock diets, farmers can not only enhance the productivity of their animals but also improve the marketability and value of their products.

### 4.5 Role in Manure Management

Managing manure in livestock and poultry production is essential for sustainable farming, as improper handling can cause environmental pollution and health issues. Biochar offers a beneficial solution for manure management by reducing harmful gas emissions like ammonia, methane and hydrogen sulphide through its porous structure, which adsorbs these gases and improves air quality around livestock facilities (Sethupathi et al. 2017; Hammerschmiedt et al. 2022; Chen et al. 2024). Furthermore, biochar aids in nutrient retention by stabilizing essential nutrients such as nitrogen, phosphorus and potassium within manure, thereby enhancing its value as an organic fertilizer and promoting soil health. Biochar also reduces pathogens like *E. coli* and *Salmonella*, making manure safer for application (Rosman and Jamaludin 2023). The researchers concluded that the addition of biochar to the soil sequestered large amounts of highly stable carbon, reduced N_2_O emissions, increased CO_2_ emissions from the soils and decreased the rates of CO_2_ emissions following the addition of manure (Rogovska et al. 2011). In a study, the application of cornstalk biochar as a carrier for microbial agents during the composting of laying hen manure demonstrated a significant reduction in NH_3_ emissions, with the separate load immobilized mixed bacteria group achieving the highest reduction of 22.61% (Zhang et al. 2020). This approach not only decreased nitrogen losses but also enhanced microbial activity and seed germination rates, indicating that biochar can effectively mitigate NH_3_ production while promoting beneficial biological processes in composting (Zhang et al. 2020). Furthermore, another study evaluated NaOH‐modified biochar's effect on NH_3_ and H_2_S emissions from laying hens’ manure during a 44‐day composting process (Cao et al. 2024). It found that the modified biochar reduced NH_3_ and H_2_S emissions by 40.63% and 77.78%, respectively, due to its enhanced adsorption properties. Changes in the microbial community also aided in converting H_2_S to stable sulphate, highlighting the biochar's potential as an effective deodorizer in composting (Cao et al. 2024). These findings highlight the significance of biochar in improving soil quality and managing greenhouse gas emissions (Rogovska et al. 2011). Its addition improves the physical properties of manure by managing moisture levels and enhancing aeration, thus minimizing odour and emissions. Furthermore, Chen et al. (2017) investigated multiple biochar types (cornstalk, bamboo, woody, layer manure and coir) for their efficacy in mitigating gaseous emissions (NH_3_ and CH_4_) during layer hen manure composting. Results showed that cornstalk biochar was particularly effective, reducing NH_3_ emissions by 24.8% and CH_4_ emissions by 26.1%, attributed to its high sorption capacity and favourable physiochemical properties (Chen et al. 2017). Additionally, NaOH‐modified biochar significantly decreased NH_3_ and H_2_S emissions by 40.63% and 77.78%, respectively, enhancing the potential for odour reduction in aerobic composting processes (Chen et al. 2017). In summary, biochar enhances manure quality and safety, supporting sustainable farming practices while effectively addressing the challenges in livestock and poultry production.

### 4.6 Effects of Biochar on Laying Hens

**FIGURE 5:**
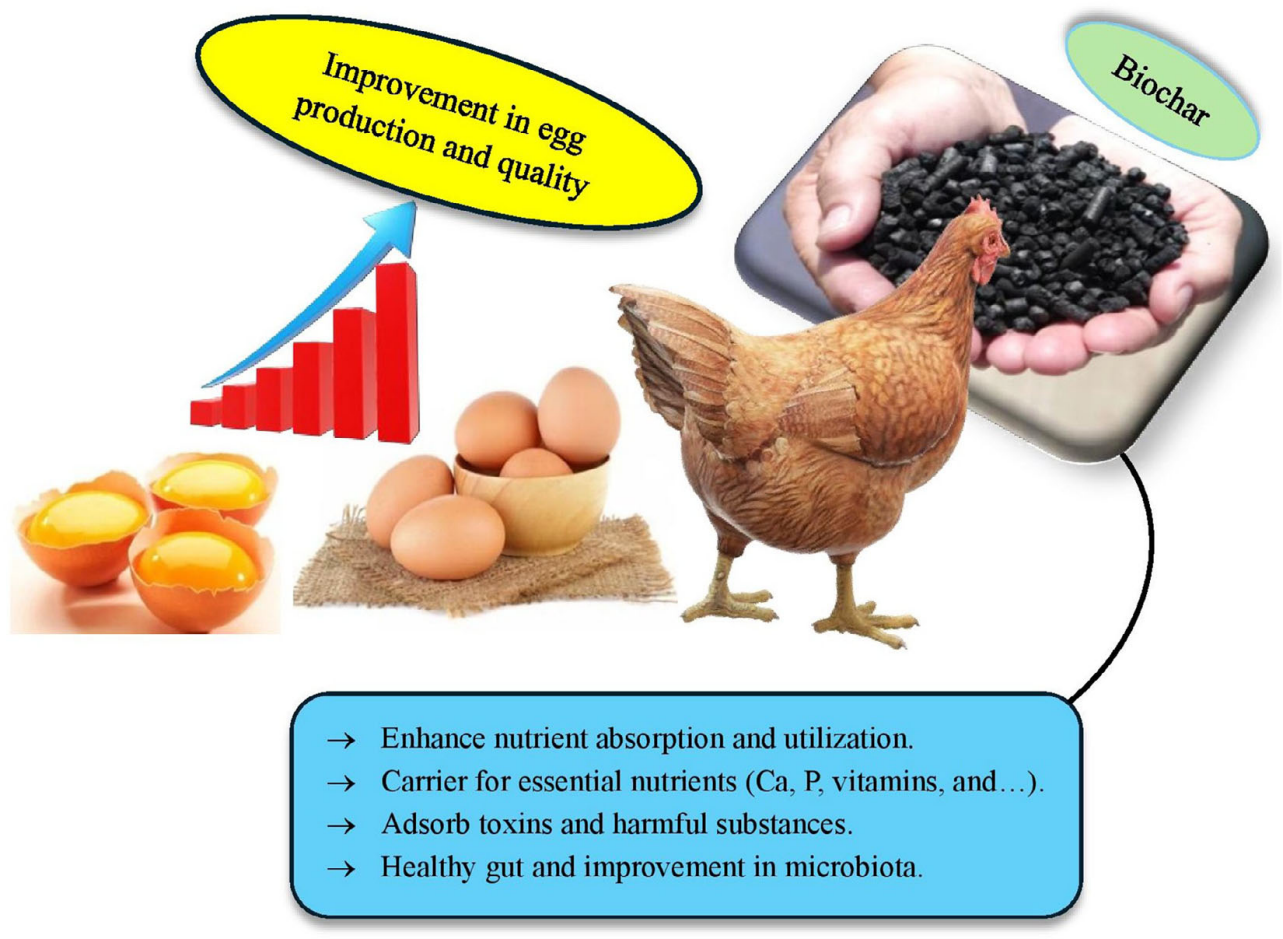
Effects of biochar on egg production and quality.

The use of biochar in poultry production has demonstrated significant potential to enhance both egg production and quality, making it a valuable tool for improving the productivity and profitability of poultry farming. One of the primary ways biochar contributes to improved egg production is through its ability to enhance nutrient absorption and utilization. The porous structure of biochar acts as a carrier for essential nutrients, such as calcium, phosphorus and vitamins, which are critical for egg formation. By ensuring a steady supply of these nutrients, biochar supports optimal reproductive performance in laying hens, leading to increased egg production rates. For example, the addition of biochar to the diet of laying hens enhanced egg quality and improved laying performance, while also showing positive effects on reducing ammonia and volatile organic compound emissions (Kalus et al. 2020). In another study, the inclusion of biochar in the diets of Bond Brown Layer pullets led to significantly improved feed conversion ratios compared to controls. The application of 2% biochar also resulted in enhanced egg weight and production performance, suggesting that biochar may function as a detoxifier and positively influence gut health, thereby optimizing feed efficiency in poultry (Prasai et al. 2017). In addition to boosting egg production, biochar plays a crucial role in improving egg quality. Egg quality is influenced by factors such as shell strength, yolk colour and nutritional content, all of which can be enhanced through the inclusion of biochar in poultry diets. For instance, biochar's high mineral content, particularly calcium, contributes to stronger eggshells, reducing the risk of breakage and improving the overall quality of eggs. In a study, incorporating biochar into the diets of laying hens, even at full substitution of mineral supplements, showed no detrimental effects on egg quality or hen performance (Ahmadi et al. 2023). The findings suggest that biochar is a viable alternative in poultry diets, offering a sustainable option without compromising productivity or egg quality metrics (Ahmadi et al. 2023). Furthermore, biochar's ability to adsorb toxins and harmful substances, such as mycotoxins, helps maintain the health of laying hens, ensuring that eggs are free from contaminants and safe for consumption (Nair et al. 2023). Biochar's impact on egg quality also extends to its influence on yolk colour and nutritional value (Kalus et al. 2020). The carotenoids and other pigments present in feed are responsible for the vibrant colour of egg yolks, which is an important quality parameter for consumers (Dansou et al. 2023). Biochar's adsorption properties may help protect these pigments from degradation in the digestive tract, resulting in richer yolk colour. Additionally, biochar's ability to enhance nutrient absorption ensures that eggs are more nutrient‐dense, with higher levels of essential vitamins and minerals, such as vitamin E and omega‐3 fatty acids. Another important aspect of biochar's role in improving egg production and quality is its ability to modulate the gut environment. A healthy gut is essential for efficient nutrient absorption and overall bird health, both of which are critical for optimal egg production. Biochar's porous structure provides a habitat for beneficial microorganisms, promoting a balanced gut microbiota (Li et al. 2023). This microbial balance supports efficient digestion and nutrient utilization, ensuring that laying hens receive the nutrients they need for consistent egg production (Dai et al. 2022). In summary, biochar's ability to improve egg production and quality is multifaceted, involving enhanced nutrient absorption, detoxification of harmful substances, modulation of gut health and protection of essential pigments. These benefits collectively contribute to higher egg production rates, stronger eggshells, richer yolk colour and more nutrient‐dense eggs. By incorporating biochar into poultry diets, farmers can enhance the productivity and quality of their layer operations, meeting consumer demand for high‐quality eggs while improving the sustainability of their farming practices. Effects of biochar on egg production and quality are shown in Figure 5. As shown in the figure, the inclusion of biochar in the diet of laying hens can enhance egg production and improve egg quality by increasing the absorption and consumption of nutrients, facilitating the transport of essential minerals (such as calcium and phosphorus), adsorbing toxins and harmful substances, improving the gut microbiota and promoting overall gut health.

## 5 Other Impacts of Biochar

### 5.1 Environmental Benefits

**FIGURE 6:**
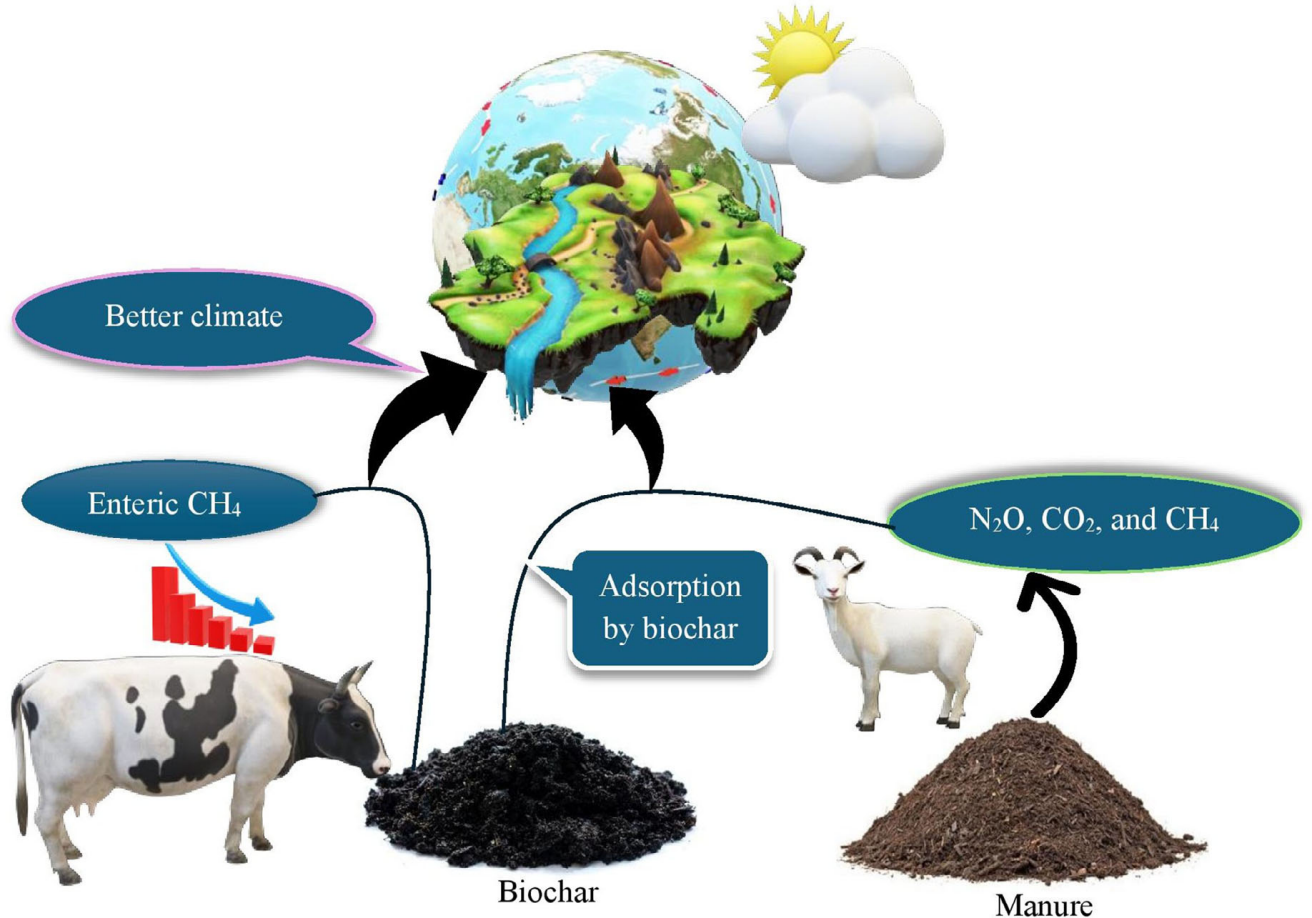
Effects of biochar in mitigating climate changes.

The application of biochar not only enhances animal health and productivity but also represents a strategic approach to combat climate change and promote sustainable farming practices. The application of biochar in livestock and poultry production extends beyond improving animal health and productivity; it also offers significant environmental benefits that contribute to sustainable farming practices. One of the most notable environmental advantages of biochar is its ability to sequester carbon and mitigate climate change (Shoudho et al. 2024). Biochar is produced through pyrolysis, a process that converts organic biomass into stable carbon structures resistant to microbial decomposition. When applied to soils or used in animal production systems, biochar locks away carbon for hundreds to thousands of years, reducing the amount of CO_2_ released into the atmosphere (Afshar and Mofatteh 2024). This carbon sequestration potential makes biochar a valuable tool for offsetting greenhouse gas emissions and combating global warming (Afshar and Mofatteh 2024). In addition to carbon sequestration, biochar plays a crucial role in reducing greenhouse gas emissions from livestock and poultry operations (Chen et al. 2024). Methane and N_2_O, two potent greenhouse gases, are by‐products of manure decomposition and enteric fermentation in ruminants (Eckard et al. 2010). Biochar's porous structure and adsorption capacity enable it to capture these gases, reducing their release into the atmosphere. For example, when added to manure, biochar can adsorb methane and N_2_O, mitigating their contribution to climate change (Shoudho et al. 2024). Similarly, in ruminant diets, biochar has been shown to reduce methane emissions by modulating microbial activity in the digestive tract (Winders et al. 2019). Biochar also contributes to environmental sustainability by improving waste management and resource efficiency. The production of biochar utilizes agricultural and organic waste materials, such as crop residues, manure and wood chips, which might otherwise be burned or left to decompose, releasing CO_2_ and other pollutants. By converting these waste materials into biochar, farmers can reduce waste, minimize pollution and create a valuable product that enhances soil health and animal production. This circular approach aligns with the principles of a circular economy, promoting resource efficiency and reducing the environmental footprint of farming operations. Additionally, biochar contributes to the improvement of water quality by retaining essential nutrients and agrochemicals in soil, thereby promoting their availability for plant uptake and reducing environmental pollution (Pandian et al. 2024; Wang and Wang 2019). When applied to soils, biochar enhances nutrient retention, preventing the leaching of nitrogen and phosphorus into water bodies (Hossain et al. 2020). This reduces the risk of eutrophication, a process that leads to algal blooms, oxygen depletion and the degradation of aquatic ecosystems. Biochar's ability to mitigate water runoff and soil erosion makes it particularly valuable in water‐scarce regions or areas with prolonged droughts and limited irrigation (Kabir et al. 2023). In summary, biochar's environmental benefits are far‐reaching, encompassing carbon sequestration, reduction of greenhouse gas emissions, improved waste management and protection of water quality. By integrating biochar into livestock and poultry production systems, farmers can contribute to climate change mitigation, enhance resource efficiency and promote sustainable agricultural practices. These environmental advantages underscore the potential of biochar as a key component of eco‐friendly farming systems. The effects of biochar in mitigating climate changes are shown in Figure 6. As illustrated in the figure, the addition of biochar to manure results in the adsorption of three major gases: methane, N_2_O and CO_2_. This process prevents their release into the atmosphere, thereby reducing air pollution caused by greenhouse gases. Additionally, feeding ruminants (Figure 6) with biochar can reduce enteric methane production, ultimately contributing to a decrease in methane emissions from the animals into the atmosphere. Therefore, biochar can play a significant role in mitigating climate change and improving air quality.

### 5.2 Carbon Sequestration and Other Effects on Soil

**FIGURE 7:**
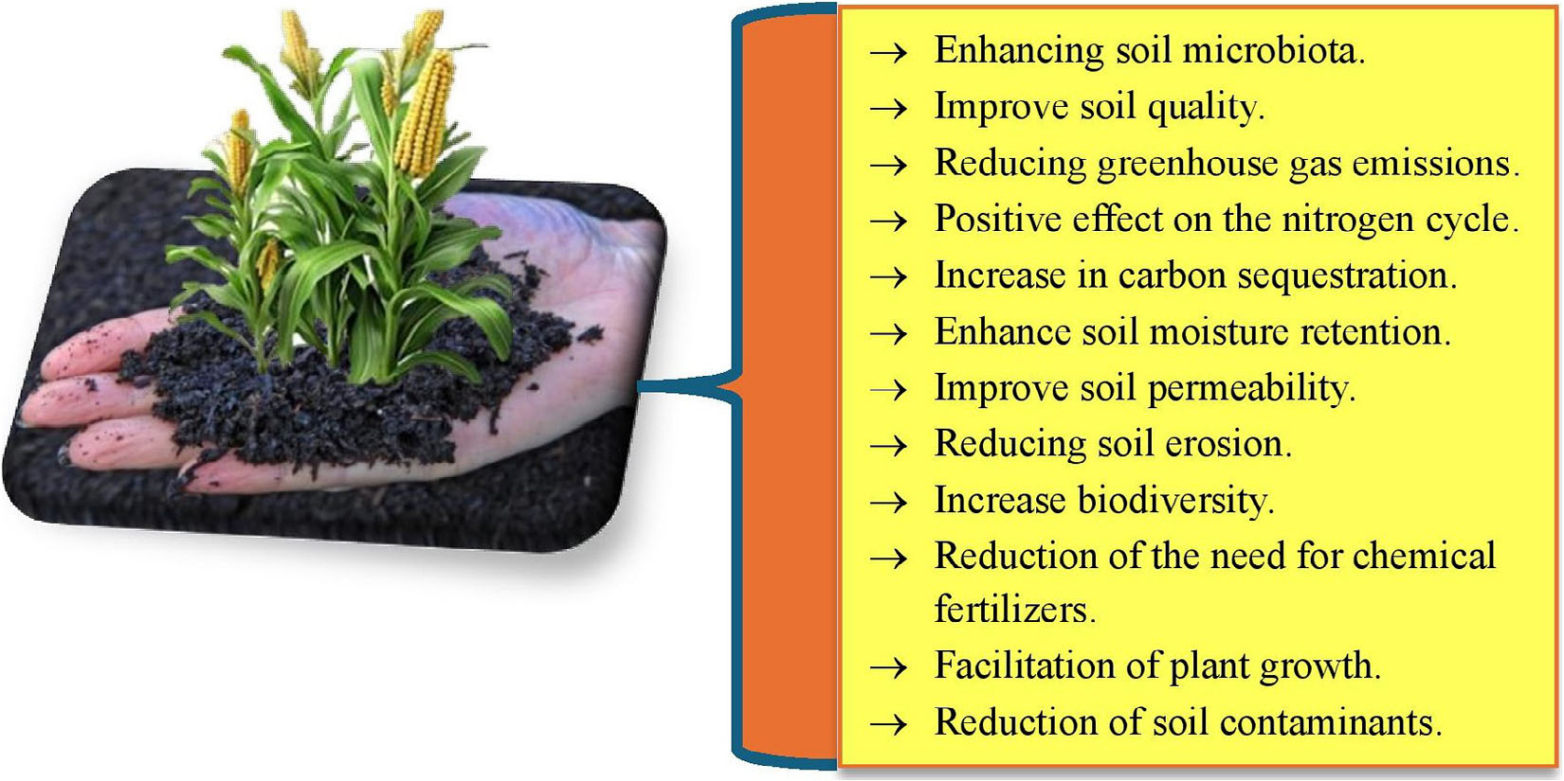
Effects of biochar on carbon sequestration and other characteristics of soil.

Biochar has emerged as a powerful tool in the fight against climate change, primarily due to its ability to sequester carbon and reduce greenhouse gas emissions (Kabir et al. 2023). The process of producing biochar, known as pyrolysis, involves heating organic biomass, such as agricultural residues, wood or manure, in the absence of oxygen. This process converts the carbon in biomass into a stable, solid form that is resistant to microbial decomposition (Afshar and Mofatteh 2024). Unlike organic matter that decomposes and releases CO_2_ back into the atmosphere, biochar can retain carbon for hundreds to thousands of years when incorporated into soils or used in animal production systems (Nayak et al. 2022; Shoudho et al. 2024). This long‐term carbon storage capability makes biochar a highly effective strategy for mitigating climate change by reducing the concentration of CO_2_ in the atmosphere. In addition to its direct carbon sequestration potential, biochar contributes to climate mitigation by reducing emissions of other greenhouse gases, such as methane and nitrous oxide, which are significantly more potent than CO_2_ in terms of their global warming potential (Kalu et al. 2022). In livestock and poultry production, methane is produced during enteric fermentation in ruminants and the anaerobic decomposition of manure, whereas nitrous oxide is released from manure management and soil applications (Zhou et al. 2007). Biochar's porous structure and adsorption capacity enable it to capture these gases, preventing their release into the atmosphere. Biochar added to manure adsorbs methane and nitrous oxide, reducing emissions and lowering farming's carbon footprint (Shrestha et al. 2023). Moreover, biochar's application in soils enhances its climate mitigation potential by improving soil health and promoting carbon storage (Velichkova et al. 2024). When biochar is added to soils, it not only sequesters carbon but also enhances soil organic carbon levels by providing a stable carbon framework that supports the formation of soil aggregates (Li et al. 2024). These aggregates protect organic matter from decomposition, further increasing the soil's capacity to store carbon. Additionally, biochar improves soil fertility and water retention, leading to increased plant growth and biomass production, which, in turn, captures more CO_2_ from the atmosphere through photosynthesis (Yadav et al. 2023). The production of biochar itself also contributes to climate mitigation by utilizing waste biomass that might otherwise be burned or left to decompose, both of which release CO_2_ and other pollutants. By converting this biomass into biochar, farmers can reduce waste, minimize emissions and create a valuable product that supports sustainable agriculture. This circular approach aligns with global efforts to transition to a low‐carbon economy and reduce reliance on fossil fuels. In summary, biochar's role in carbon sequestration and climate mitigation is multifaceted, involving long‐term carbon storage, reduction of greenhouse gas emissions, enhancement of soil carbon levels and utilization of waste biomass. By integrating biochar into livestock and poultry production systems, farmers can play a significant role in combating climate change while improving the sustainability and productivity of their operations. This makes biochar a key component of climate‐smart agriculture and a promising solution for achieving global climate goals. Effects of biochar on carbon sequestration and other characteristics of soil are shown in Figure 7. As explained in the figure, the use of biochar in agriculture and horticulture has gained attention as one of the methods for carbon sequestration in soil. The effects of biochar on carbon sequestration in soil can be explained in several ways. For instance, biochar is effective in increasing soil carbon storage. Due to its porous structure, biochar can store carbon in the soil and slow down its decomposition process, thereby extending the duration of carbon storage. Additionally, biochar improves soil quality by enhancing its physical, chemical and biological properties. These improvements include increased water retention capacity, reduced soil erosion and enhanced microbial activity, all of which contribute to carbon sequestration. Biochar also plays a role in reducing greenhouse gas emissions. By using biochar, the release of greenhouse gases such as methane and nitrous oxide can be mitigated, ultimately helping to lessen the impacts of climate change. Furthermore, biochar positively influences the nitrogen cycle by improving nitrogen management in the soil. As nitrogen is a key factor for plant growth, providing adequate nitrogen through biochar can enhance plant performance, increase carbon dioxide uptake from the atmosphere and promote its storage in the soil. Beyond these general nitrogen cycle improvements, specific studies demonstrate that biochar creates favourable microenvironments for nitrogen‐cycling bacteria (Ding and Yu 2024). Research shows biochar enhances nitrogen fixation in root nodules (Xiu et al. 2021), promotes nitrification (Guo et al. 2021) and reduces nitrogen leaching (Liu et al. 2017). Its porous structure and redox‐active surfaces also modulate denitrification processes, supporting populations of nitrifying and denitrifying bacteria (Liu et al. 2016) while simultaneously lowering N_2_O (Ding and Yu 2024). These microbial‐mediated transformations make biochar particularly effective for optimizing soil nitrogen dynamics. Biochar is also effective in regulating soil temperature and moisture. It helps maintain soil moisture and, under certain conditions, controls temperature, which can enhance microbial activity and further improve carbon sequestration. The use of biochar as a strategy for carbon sequestration in soil can play a significant role in sustainable resource management and climate change mitigation. Moreover, biochar promotes microbial activity in the soil by providing an ideal environment for the growth and development of microorganisms. This increased microbial activity can enhance the decomposition of organic matter and improve nutrient availability for plants. Biochar facilitates plant growth by slowly releasing nutrients, which can lead to better and faster plant development, particularly in nutrient‐poor soils. It also contributes to reducing soil pollution by adsorbing and stabilizing certain soil contaminants, such as heavy metals and hazardous organic compounds, thereby lowering the risk of pollution and improving soil quality. Additionally, biochar reduces the need for chemical fertilizers by increasing the efficiency of nitrogen and other nutrient uptake, which not only lowers costs but also reduces pollution. Biochar enhances soil biodiversity by improving soil structure and quality, which can increase biodiversity within the soil. This diversity can contribute to the stability of agricultural ecosystems and help control pests and diseases. Furthermore, biochar is effective in reducing soil erosion by improving soil structure and its physical properties, which helps preserve nutrient‐rich topsoil layers. Finally, biochar improves soil permeability due to its porous structure, enhancing the infiltration of water and air. This can prevent waterlogging and improve soil respiration. These positive attributes and effects of biochar can contribute to more sustainable agriculture and increased crop productivity.

### 5.3 Reduction of Enteric CH_4_ in Ruminants

The greenhouse gas CH_4_ is a significant contributor to climate change, with enteric fermentation in ruminants being one of its primary sources. Ruminants, such as cattle and sheep, produce CH_4_ as a by‐product of microbial fermentation during digestion, specifically as an end product of feed fermentation in the rumen (Tseten et al. 2022). Recent research has shown that biochar, a carbon‐rich material produced through the pyrolysis of organic materials, can effectively mitigate methane emissions in these animals (Burezq and Khalil 2025). Biochar reduces enteric methane emissions through multiple mechanisms: Its porous structure modulates the rumen microbiome, suppressing methanogenic archaea while promoting hydrogen‐utilizing bacteria that favour alternative metabolic pathways. Additionally, biochar shifts microbial populations towards fermentative bacteria that produce volatile fatty acids, diverting hydrogen from methanogenesis. Furthermore, it enhances feed efficiency by improving nutrient absorption, which increases animal productivity (e.g., meat and milk yield) and ultimately reduces methane emissions per unit of output (Martinez‐Fernandez et al. 2024; Lind et al. 2024). Research indicates that incorporating biochar into ruminant diets can result in a remarkable decrease in enteric methane emissions, with some studies showing reductions of up to 22% (Leng et al. 2012). Moreover, biochar's effects on gut health cannot be overlooked. Its absorptive and surface properties can reduce the presence of pathogenic bacteria in the rumen, which promotes healthier microflora. A healthier rumen environment can lead to increased production of short‐chain fatty acids, which are more efficient energy sources, thus lowering the amount of substrate available for methanogenesis. Furthermore, the type of biochar used plays a critical role in its efficacy. Studies indicate that biochar derived from different feedstocks, such as agricultural waste or forest residues, can produce varied effects on methane reduction (Teoh et al. 2019; Tamayao et al. 2021; Sperber et al. 2022). Therefore, understanding the physicochemical properties of biochar is essential to maximize its benefits in ruminant management. In general, the reduction of enteric methane emissions in ruminants through the implementation of biochar presents a promising strategy for addressing climate change while improving livestock productivity. Ongoing research is necessary to explore the long‐term impacts of biochar on ruminant health and its implications for sustainable agriculture. As we strive for a more sustainable future, integrating biochar into ruminant diets could be a pivotal step in reducing greenhouse gas emissions in the livestock sector.

## 6 Challenges and Limitations of Biochar Use in Animal Production

### 6.1 Variability in Biochar Quality and Composition

One of the most significant challenges in the widespread adoption of biochar in animal production is the variability in its quality and composition. Biochar is produced from a wide range of feedstocks, including wood, crop residues, manure and other organic materials, each with distinct chemical and physical properties (Tomczyk et al. 2020; Seow et al. 2022). The pyrolysis process itself, including factors such as temperature, heating rate and duration, further influences the characteristics of the final product (Zhang et al. 2024). As a result, biochar can vary widely in terms of its carbon content, porosity, surface area, mineral composition and stability. This variability poses challenges for standardizing its use in animal diets and manure management systems, as the effectiveness of biochar can differ significantly depending on its properties. For instance, biochar produced at lower pyrolysis temperatures may retain more organic compounds and have a higher content of functional groups, making it more reactive but less stable (Yaashikaa et al. 2020). Conversely, biochar produced at higher temperatures tends to have a more stable carbon structure (characterized by more aromatic structures) and greater porosity but may lack certain functional groups that enhance its adsorption capacity and stability (Tan et al. 2021; Tsolis and Barouchas 2023). This inconsistency in quality can lead to unpredictable outcomes when biochar is used as a feed additive or soil amendment, making it difficult for farmers to achieve consistent results. Another issue related to variability is the potential presence of contaminants in biochar, such as heavy metals, polycyclic aromatic hydrocarbons or residual chemicals from treated feedstocks (Hilber et al. 2017; Luo et al. 2020). These contaminants can pose risks to animal health and the environment if not properly managed. For example, biochar derived from industrial waste or treated wood may contain harmful substances that could accumulate in animal tissues or leach into soil and water systems. The ecological concerns associated with the production of preservative‐treated, lignocellulosic biochar warrant attention, as minute quantities of toxic heavy metals, especially arsenic, may volatilize into the atmosphere during pyrolysis. A significant proportion of heavy metals accumulates in biochar at concentrations exceeding those found in typical or contaminated soils (Huang et al. 2018; Kim et al. 2020). Consequently, these metals can leach into the soil matrix and disrupt ecological balance, particularly affecting biomass proliferation (Li et al. 2019). The bioavailability of heavy metals that are assimilated and translocated into biomass can induce metabolic dysfunctions, inhibit mitotic processes, trigger cellular apoptosis or modify the structural integrity and functionality of various cellular membranes and enzymes (Dadrasnia et al. 2013; Singh et al. 2013). Ensuring the safety and quality of biochar requires rigorous testing and certification, which can be resource‐intensive and may not be feasible for all producers. Furthermore, the lack of standardized guidelines for biochar production and application exacerbates the challenges associated with variability. Without clear standards, farmers and producers may struggle to select the appropriate type of biochar for specific applications, leading to suboptimal results or unintended consequences. Developing standardized protocols for biochar production, characterization and use is essential for maximizing its benefits and minimizing risks in animal production systems. In summary, the variability in biochar quality and composition presents a significant challenge for its use in animal production. Differences in feedstock, pyrolysis conditions and potential contaminants can affect the performance and safety of biochar, making it difficult to achieve consistent and reliable outcomes. Addressing these challenges requires further research, standardization and the development of clear guidelines to ensure that biochar can be effectively and safely integrated into livestock and poultry farming systems.

### 6.2 Potential Risks and Safety Concerns

Although biochar offers numerous benefits for animal production, its use is not without potential risks and safety concerns that must be carefully addressed. One of the primary concerns is the presence of contaminants in biochar, which can vary depending on the feedstock and production process (Kabir et al. 2023). For example, biochar derived from industrial waste, treated wood or certain agricultural residues may contain heavy metals, polycyclic aromatic hydrocarbons or other harmful substances (Hilber et al. 2017; Luo et al. 2020). If these contaminants are not adequately removed or neutralized, they can accumulate in animal tissues, posing risks to animal health and potentially entering the food chain. This raises concerns about food safety and consumer health, particularly in cases where biochar is used as a feed additive. Another safety concern is the potential for biochar to alter the pH of the digestive tract or soil when used in large quantities (Hossain et al. 2020; Osman et al. 2022). However, a study revealed that the inclusion of 30 g/kg biochar in broiler diets did not significantly affect the pH levels of various digestive organs, including the crop, proventriculus, gizzard, ileum or cecum (Goiri et al. 2021). Despite the observed changes in performance and fatty acid profiles, biochar maintained the pH stability within the digestive system during the feeding period (Goiri et al. 2021). Furthermore, in a study conducted on Kermanian ram lambs, the ruminal pH was not affected by biochar prepared from walnut shells, pistachio by‐products and chicken manure (Mirheidari et al. 2020). Nevertheless, biochar is often alkaline, and its application in excessive amounts can lead to an increase in pH levels, which may disrupt the gut microbiota in animals or affect soil microbial communities (Zhu et al. 2023). In livestock, an imbalanced gut pH can impair digestion, reduce nutrient absorption and increase susceptibility to diseases. Similarly, in soil, an elevated pH can negatively impact plant growth and nutrient availability, counteracting the intended benefits of biochar as a soil amendment (Gunarathne et al. 2017; Barrow and Hartemink 2023). The physical properties of biochar also pose potential risks, particularly its fine particulate nature (Thomas 2021). Inhalation of biochar dust by animals or workers can cause respiratory issues, such as irritation or inflammation of the airways (Pinelli et al. 2024). This is especially relevant in poultry houses or confined livestock facilities where biochar is mixed with feed or litter. Proper handling and application methods, such as pelletizing biochar or using dust suppression techniques, are necessary to minimize these risks (Mohammadi 2021). Additionally, the long‐term effects of biochar on animal health and the environment are not yet fully understood. Although biochar is generally considered stable and resistant to degradation, its interaction with the digestive system, soil and microbial communities over extended periods requires further investigation. For instance, the gradual release of adsorbed substances or the potential for biochar to alter microbial dynamics in ways that could harm animal health or ecosystem balance remains a topic of ongoing research. In summary, although biochar holds great promise for improving animal production systems, its use is accompanied by potential risks and safety concerns that must be carefully managed. Contaminants, pH alterations, respiratory hazards and uncertainties about long‐term effects highlight the need for rigorous testing, standardized guidelines and responsible application practices. Addressing these concerns is essential to ensure that biochar can be safely and effectively integrated into livestock and poultry farming, maximizing its benefits while minimizing potential risks.

### 6.3 Economic Feasibility and Scalability

The economic feasibility and scalability of biochar use in animal production are critical factors that influence its adoption and widespread implementation. One of the primary challenges is the cost associated with biochar production, which includes expenses related to feedstock acquisition, pyrolysis equipment, energy consumption and labour. Although biochar can be produced from low‐cost or waste materials, such as agricultural residues or manure, the initial investment in pyrolysis technology and infrastructure can be prohibitive for small‐scale farmers or those in resource‐limited settings (Kumari et al. 2023). Biochar demonstrates promising economic potential in animal production systems when considering both direct and indirect benefits. Case studies reveal that while initial production costs pose a barrier, long‐term advantages, including improved feed efficiency, reduced mortality and lower veterinary expenses, can offset these investments. Successful implementations highlight significant returns through enhanced productivity and operational savings, particularly when utilizing low‐cost agricultural waste as feedstock. The economic viability further improves when integrated with carbon credit programmes or government incentives. However, widespread adoption requires addressing upfront capital costs through innovative solutions like shared pyrolysis facilities and standardized production methods to ensure consistent quality and cost‐effectiveness across different farming scales. Additionally, the variability in biochar quality and the need for consistent production standards further complicate cost‐effectiveness, as higher quality biochar often requires more controlled and expensive production processes. Another economic consideration is the return on investment for farmers. Although biochar has demonstrated benefits such as improved feed efficiency, enhanced animal health and reduced environmental impacts, the financial gains from these benefits may not always offset the upfront costs (Burezq and Khalil 2025). For example, the improved growth performance or reduced mortality rates attributed to biochar may take time to translate into measurable economic returns, making it difficult for farmers to justify the initial expenditure. Furthermore, the lack of established markets for biochar and its by‐products can limit its economic viability, as farmers may struggle to find buyers or additional revenue streams. Scalability is another significant challenge for biochar use in animal production. Although small‐scale applications have shown promising results, scaling up biochar production and use to meet the demands of large commercial operations presents logistical and technical hurdles. Large‐scale pyrolysis facilities require significant capital investment, reliable feedstock supply chains and efficient distribution networks (Patel et al. 2019). Additionally, the integration of biochar into existing animal production systems may require modifications to feeding equipment, manure management practices or farm infrastructure, further increasing costs and complexity. Despite these challenges, there are opportunities to improve the economic feasibility and scalability of biochar. For instance, government subsidies, carbon credit programmes or incentives for sustainable farming practices could help offset production costs and encourage adoption (Raina et al. 2024). Collaborative efforts between researchers, industry stakeholders and policymakers can also drive innovation in biochar production technologies, making them more affordable and accessible. Furthermore, educating farmers about the long‐term benefits of biochar, such as improved soil health, reduced fertilizer costs and enhanced animal productivity, can help build confidence in its economic value. In summary, although biochar holds significant potential for improving animal production systems, its economic feasibility and scalability remain key challenges. Addressing these issues requires a combination of technological innovation, financial incentives and stakeholder collaboration to make biochar a viable and scalable solution for sustainable farming. By overcoming these barriers, biochar can play a transformative role in enhancing the productivity and sustainability of livestock and poultry production worldwide.

### 6.4 Lack of Standardized Guidelines and Regulations

The integration of biochar into livestock and poultry production is hindered by the lack of standardized guidelines and regulations. This inconsistency affects biochar quality, application methods and safety protocols, which can lead to unpredictability in its effectiveness and farmer acceptance. Without clear regulations, producers may struggle to identify appropriate feedstocks and pyrolysis conditions for producing high‐quality biochar. Variability in biochar properties, such as nutrient content and pH levels (Prasad et al. 2019), makes it difficult for farmers to predict performance in various applications, potentially resulting in suboptimal outcomes. Additionally, the absence of standards raises food safety and environmental concerns, particularly when biochar is produced from contaminated feedstocks. These issues may deter farmers from adopting biochar due to concerns about impacts on animal welfare and product safety. The lack of regulations also hinders research and development, making it challenging to conduct comparative studies or establish best practices for biochar use in livestock. Addressing these challenges requires collaboration among researchers, industry representatives and policymakers to develop comprehensive guidelines. Such efforts should focus on establishing minimum quality standards and implementing safety assessments to enhance the credibility of biochar in livestock production. In conclusion, standardized guidelines are crucial for the effective use of biochar in animal farming and addressing these gaps can promote safer practices and broader acceptance in sustainable agriculture.

## 7 Future Prospects and Research Directions

### 7.1 Optimization of Biochar Production for Animal Applications

The future of biochar in livestock and poultry production holds immense potential, particularly in optimizing its production processes to enhance its efficacy and safety in animal applications. As interest in biochar grows, research must focus on refining pyrolysis techniques to produce biochar with tailored properties that meet the specific needs of animal nutrition and health. One promising avenue for optimization is the careful selection of feedstocks. Different organic materials, such as agricultural residues, manure and forestry by‐products, can yield biochar with varying nutrient profiles and physicochemical characteristics (Ebrahimi et al. 2024). Future studies should investigate the impact of diverse feedstocks on biochar's performance as a feed additive and soil amendment. By identifying the most beneficial feedstock combinations, researchers can maximize nutrient content and adsorption capacity, thereby enhancing the overall effectiveness of biochar in animal production. Additionally, the pyrolysis parameters, including temperature, heating rate and residence time, play a critical role in determining the final properties of biochar (de Oliveira Paiva et al. 2024). Future research should aim to establish optimal pyrolysis conditions that not only enhance biochar's stability and nutrient retention but also minimize the formation of potentially harmful compounds. By conducting systematic studies on how different pyrolysis settings affect biochar characteristics, scientists can develop guidelines for producing high‐quality biochar tailored for specific animal applications. Moreover, exploring innovative post‐production treatments could further enhance biochar's functionality. Techniques such as chemical activation or blending with other natural additives may improve biochar's adsorption properties and nutrient availability. Research in this area can lead to the development of biochar formulations that are more effective in promoting gut health, reducing pathogens and improving overall animal performance. Another critical research direction is the long‐term impact of biochar use in animal systems. Investigating how biochar interacts with animal physiology, gut microbiota and manure management over extended periods will provide valuable insights into its sustainability and safety. Understanding these interactions can guide recommendations for biochar usage, ensuring it aligns with best practices for animal welfare and environmental stewardship. In summary, the optimization of biochar production for animal applications is a promising area for future research. By focusing on feedstock selection, pyrolysis conditions, post‐production enhancements and long‐term impacts, the agricultural community can unlock the full potential of biochar as a sustainable tool in livestock and poultry production. This multifaceted approach will not only improve animal health and productivity but also contribute to more sustainable agricultural practices.

### 7.2 Integration With Precision Livestock Farming (PLF)

The integration of biochar into PLF represents a transformative approach that leverages technology to enhance animal production while promoting sustainability (Assimakopoulos et al. 2025). PLF utilizes advanced monitoring systems and data analytics to optimize animal care, feed management and overall farm efficiency. By incorporating biochar into these systems, farmers can achieve more precise control over animal health and environmental impacts (Norton et al. 2019). One of the key benefits of biochar in PLF is its ability to improve feed quality. By analysing the nutritional requirements of livestock, biochar can be tailored to complement specific diets. Its unique properties allow it to adsorb toxins and enhance nutrient availability, which can be monitored through real‐time data collection. This integration ensures that animals receive optimal nutrition, leading to improved growth rates and overall health. Moreover, biochar's role in enhancing gut health can be effectively monitored through precision farming technologies. Sensors and digital platforms can track changes in animal behaviour, feed intake and health metrics, providing valuable insights into the effectiveness of biochar as a feed additive (Akintan et al. 2025). This data‐driven approach allows farmers to make informed decisions about biochar application, ensuring optimal contributions to animal welfare and productivity. In addition to its nutritional benefits, biochar significantly improves manure management within precision livestock systems. By incorporating biochar into manure treatment processes, farmers can reduce odours and greenhouse gas emissions. Advanced monitoring technologies can measure the effectiveness of biochar in mitigating these emissions, providing quantifiable evidence of its environmental benefits. This not only helps in complying with regulatory standards but also enhances the sustainability of livestock operations. Furthermore, the integration of biochar with PLF can facilitate better resource management. By using data analytics to assess the impact of biochar on soil health and nutrient retention, farmers can optimize their fertilization strategies. This ensures that nutrients are used efficiently, reducing waste and minimizing environmental impact. The potential for biochar to improve animal welfare is also noteworthy. Through precision monitoring, farmers can observe the effects of biochar on stress levels, disease resistance and overall animal behaviour. By creating a healthier living environment, biochar can contribute to better animal performance and lower veterinary costs, ultimately benefiting farm profitability (Graves et al. 2022). In summary, the integration of biochar with PLF offers a holistic approach to enhancing animal production and sustainability. By combining advanced technology with the multifunctional properties of biochar, farmers can optimize nutrition, improve manure management and promote animal welfare. This innovative synergy not only addresses current challenges in livestock production but also paves the way for a more sustainable future in agriculture.

### 7.3 Exploration of Synergistic Effects With Other Feed Additives

The exploration of synergistic effects between biochar and other feed additives presents a promising frontier in enhancing animal nutrition and health. By understanding how biochar interacts with various feed components, researchers can develop more effective combinations that optimize animal performance and welfare. This approach not only maximizes the benefits of biochar but also addresses the complex nutritional needs of livestock. One area of interest is the combination of biochar with probiotics (Benhissi et al. 2025). Probiotics are known to improve gut health and enhance nutrient absorption in animals (Ding et al. 2021). When biochar is used alongside probiotics, it may create a more favourable environment in the gastrointestinal tract, promoting the growth of beneficial microorganisms (Benhissi et al. 2025). This synergy could lead to improved digestion, better feed conversion rates and enhanced immunity, ultimately resulting in healthier animals and increased productivity (Benhissi et al. 2025). Furthermore, magnetic biochar, an advanced variant of conventional biochar, serves as an eco‐friendly, efficient and economically viable bactericidal agent, demonstrating significant antimicrobial efficacy against waterborne pathogens (Mukherjee et al. 2025). The interaction of magnetic biochar with quaternary phosphonium salt yields magnetic biochar–quaternary phosphonium salt (MBQ), a derivative with enhanced antimicrobial characteristics compared to standard biochar and magnetic biochar (Mukherjee et al. 2025). This compound effectively inhibits the growth of waterborne pathogens, including *E. coli* and *Staphylococcus aureus*. MBQ compromises bacterial membrane integrity and induces oxidative damage within microbial cells, leading to their inactivation (Mukherjee et al. 2025). Another promising avenue is the integration of biochar with natural antimicrobials or essential oils. These additives can help control pathogens in the gut, reducing antibiotics dependence in livestock production. When combined with biochar, which has its own antimicrobial properties, the effectiveness of these natural additives may be amplified. This dual action could lead to a more robust defence against harmful bacteria, improving overall gut health and reducing disease incidence in livestock. Additionally, the pairing of biochar with mineral supplements offers significant potential for synergy. Biochar has been shown to enhance the bioavailability of certain minerals, such as calcium and phosphorus, which are crucial for animal growth and development (Glaser and Lehr 2019; Alkharabsheh et al. 2021; Ahmed et al. 2024). By studying the interactions between biochar and various mineral additives, researchers can identify optimal formulations that ensure animals receive adequate nutrition while minimizing waste. This not only benefits animal health but also contributes to more sustainable farming practices by reducing nutrient runoff into the environment. Furthermore, the incorporation of biochar with fibre‐rich feed additives may enhance its effects on gut health and fermentation processes. Dietary fibre enhances gastrointestinal health through multiple mechanisms, including modulation of gut microbiota composition and metabolism, provision of energy to colonic epithelial cells, promotion of intestinal mucosa synthesis, stimulation of peristalsis and maintenance of intestinal barrier integrity (Ye et al. 2022; Han et al. 2023). When biochar is combined with high‐fibre feeds, it may improve the fermentation process in the rumen, leading to better nutrient utilization and energy production. This synergy can result in improved weight gain and feed efficiency in ruminants, making it a valuable strategy for livestock producers. In summary, the exploration of synergistic effects between biochar and other feed additives opens new avenues for improving animal nutrition and health. By leveraging the unique properties of biochar in combination with probiotics, natural antimicrobials, mineral supplements and fibre‐rich feeds, researchers can develop innovative feeding strategies that enhance livestock productivity while promoting sustainability. This integrated approach not only addresses the nutritional challenges faced by the livestock industry but also paves the way for more responsible and eco‐friendly farming practices.

### 7.4 Long‐Term Studies on Animal Health and Environmental Impacts

Long‐term studies on the effects of biochar in livestock systems are crucial for understanding its implications for animal health and environmental sustainability. These studies provide valuable insights into how biochar influences various aspects of animal well‐being, productivity and ecological balance over extended periods. One significant area of focus is the impact of biochar on animal health. Long‐term research can help identify any potential benefits or drawbacks associated with continuous biochar supplementation in diets. For instance, investigating its effects on gut microbiota diversity and function can reveal how biochar supports digestive health and nutrient absorption. Such insights are essential for developing feeding strategies that enhance animal welfare and performance. Moreover, assessing the long‐term environmental impacts of biochar application in livestock operations is vital. Biochar has the potential to improve soil health by enhancing nutrient retention and reducing erosion (Kabir et al. 2023). Longitudinal studies can measure changes in soil quality, microbial activity and carbon sequestration over time, providing a comprehensive understanding of biochar's role in promoting sustainable agricultural practices. Additionally, evaluating the persistence of biochar in manure management systems is critical. Long‐term studies can help determine how biochar affects the decomposition of organic matter and the release of greenhouse gases. Understanding these dynamics is essential for developing strategies that minimize environmental footprints while maximizing the benefits of biochar. Furthermore, such studies can also explore the economic implications of biochar use in livestock farming. By analysing production costs, feed efficiency and overall profitability over time, researchers can provide farmers with data‐driven recommendations that support both animal health and environmental stewardship. In summary, long‐term studies on biochar's effects on animal health and environmental impacts are essential for advancing our understanding of its role in sustainable livestock production. These investigations will not only inform best practices but also contribute to the development of holistic approaches that benefit animals, farmers and the environment alike.

## 8 Conclusion

The integration of biochar into livestock farming presents a unique multi‐benefit solution to enhance animal health, optimize feed efficiency and promote environmental sustainability. When combined with additives like probiotics, natural antimicrobials and mineral supplements, biochar enhances gut health, nutrient absorption and overall livestock performance. This combination not only maximizes nutritional benefits but also reduces antibiotic reliance, addressing critical concerns in modern animal husbandry. To accelerate adoption, policymakers should prioritize incentives (e.g., subsidies and carbon credits) for biochar use in agriculture, particularly in regions with high livestock emissions. Research confirms the dual benefits of biochar on animal health and environmental impacts. Continuous supplementation improves digestive health, enhances soil quality and reduces greenhouse gas emissions. Despite these benefits, challenges such as variability in biochar quality, economic feasibility and the need for standardized guidelines remain. Regulatory frameworks supporting standardized biochar production and quality control would further ensure consistent results across farming systems. Biochar's soil‐enhancing and carbon‐sequestering properties position it as a key tool in sustainable agriculture, aligning with global climate goals. Governments and agricultural extensions should integrate biochar into climate‐smart farming initiatives to maximize its scalability. Addressing these challenges is essential for widespread adoption and effective use in various agricultural systems, ensuring that biochar can be safely integrated into existing farming practices. Biochar improves feed efficiency and animal welfare economically, boosting farm profitability while promoting sustainability. Long‐term data empower farmers to adopt biochar confidently, ensuring both economic and environmental benefits. The article concludes by identifying promising research directions that could optimize biochar applications for more sustainable livestock production. In general, biochar's integration into PLF, particularly when combined with complementary additives, represents a transformative approach for sustainable agriculture. Ongoing research will help optimize biochar applications and long‐term outcomes. Multistakeholder collaborations (farmers, researchers and policymakers) are essential to mainstream biochar as a win–win solution. By leveraging its synergistic benefits, stakeholders can build a more resilient livestock industry that benefits animals, farmers and ecosystems alike.

## Author Contributions

Mohsen Kazemi is the sole author of this review article. He conducted a comprehensive literature review, synthesized information from various sources and critically analysed existing studies. All aspects of the article have been thoroughly reviewed and approved by the author.

## Ethics Statement

The author verifies that the journal's ethical standards, as detailed on the journal's author guidelines page, have been complied with. As this is a review article lacking original research data, ethical approval was not necessary.

## Conflicts of Interest

The author declares no conflicts of interest.

## Data Availability

The data used in this review article are derived from reputable sources and do not include any original data from the author.

## References

[vms370629-bib-0001] Afshar, M. , and S. Mofatteh . “Biochar for a Sustainable Future: Environmentally Friendly Production and Diverse Applications.” Results in Engineering 23: 102433.

[vms370629-bib-0002] Ahmadi, F. , M. Afsharmanesh , and M. Khajeh Bami . 2023. “Replacing Biochar With Mineral Premix and Its Interaction With Vitamin C on Laying Hen Production and Egg Quality Factors.” Animal Biotechnology 34, no. 7: 3039–3045. 36244031 10.1080/10495398.2022.2130796

[vms370629-bib-0003] Ahmadi, F. , M. Afsharmanesh , M. Salarmoini , and M. Khajeh Bami . 2022. “Investigating the Effects of Using Biochar as a Replacement for Minerals Premix in Layers Diet on Physical and Mechanical Properties of Their Egg Shells.” Biomechanism and Bioenergy Research 1, no. 1: 12–15.

[vms370629-bib-0004] Ahmadou, A. , N. Brun , A. Napoli , N. Durand , and D. Montet . 2021. “Binders Used in Feed for Their Protection Against Mycotoxins.” In Mycotoxins in Food and Beverages. CRC Press.

[vms370629-bib-0005] Ahmed, N. , L. Deng , C. Wang , et al. 2024. “Advancements in Biochar Modification for Enhanced Phosphorus Utilization in Agriculture.” Land 13, no. 5: 644.

[vms370629-bib-0006] Akintan, O. A. , K. G. Gebremedhin , and D. D. Uyeh . 2025. “Linking Animal Feed Formulation to Milk Quantity, Quality, and Animal Health Through Data‐Driven Decision‐Making.” Animals 15, no. 2: 162. 39858162 10.3390/ani15020162 PMC11758612

[vms370629-bib-0007] Alkharabsheh, H. M. , M. F. Seleiman , M. L. Battaglia , et al. 2021. “Biochar and Its Broad Impacts in Soil Quality and Fertility, Nutrient Leaching and Crop Productivity: A Review.” Agronomy 11, no. 5: 993.

[vms370629-bib-0008] Alothman, Z. A. , E. Yilmaz , M. Habila , and M. Soylak . 2015. “Solid Phase Extraction of Metal Ions in Environmental Samples on 1‐(2‐Pyridylazo)‐2‐Naphthol Impregnated Activated Carbon Cloth.” Ecotoxicology and Environmental Safety 112: 74–79. 25463856 10.1016/j.ecoenv.2014.10.032

[vms370629-bib-0009] Amalina, F. , A. S. Abd Razak , S. Krishnan , H. Sulaiman , A. W. Zularisam , and M. Nasrullah . 2022. “Biochar Production Techniques Utilizing Biomass Waste‐Derived Materials and Environmental Applications – A Review.” Journal of Hazardous Materials Advances 7: 100134.

[vms370629-bib-0010] Amean, M. A. , and T. A. Shujaa . 2020. “The Effect of Using Different Levels of Biochar on Some Performance and Digestibility of Nutrients of the Awassi Lambs.” Kirkuk University Journal for Agricultural Sciences 11, no. 3: 21–31.

[vms370629-bib-0011] Appell, M. , E. C. Wegener , B. K. Sharma , F. J. Eller , K. O. Evans , and D. L. Compton . 2023. “In Vitro Evaluation of the Adsorption Efficacy of Biochar Materials on Aflatoxin B1, Ochratoxin A, and Zearalenone.” Animals 13, no. 21: 3311. 37958067 10.3390/ani13213311 PMC10649945

[vms370629-bib-0012] Assimakopoulos, F. , C. Vassilakis , D. Margaris , K. Kotis , and D. Spiliotopoulos . 2025. “AI and Related Technologies in the Fields of Smart Agriculture: A Review.” Information 16, no. 2: 100.

[vms370629-bib-0014] Bagherpoor, Z. , J. Rezaei , and Y. Rouzbehan . 2023. “Potential of Biochar in Enhancing the Effectiveness of Probiotics Bacilli and Lactobacilli on In Vitro Microbial Populations, Hydrolytic Enzymes, and Ruminal Fermentation in Sheep.” Animal Production Research 12, no. 3: 29–47.

[vms370629-bib-0015] Barrow, N. J. , and A. E. Hartemink . 2023. “The Effects of pH on Nutrient Availability Depend on Both Soils and Plants.” Plant and Soil 487, no. 1: 21–37.

[vms370629-bib-0016] Benhissi, H. , M. Medjadbi , S. E. Charef , et al. 2025. “Probiotic‐Inoculated Biochar as a Feed Additive for Dairy Sheep: Effect on Apparent Digestibility, Microbial Protein Supply, Methane Emissions and Productive Performance.” Animal Feed Science and Technology 321: 116257.

[vms370629-bib-0017] Bolan, S. , D. Hou , L. Wang , et al. 2023. “The Potential of Biochar as a Microbial Carrier for Agricultural and Environmental Applications.” Science of the Total Environment 886: 163968. 37164068 10.1016/j.scitotenv.2023.163968

[vms370629-bib-0018] Burachevskaya, M. , T. Minkina , T. Bauer , et al. 2023. “Fabrication of Biochar Derived From Different Types of Feedstocks as an Efficient Adsorbent for Soil Heavy Metal Removal.” Scientific Reports 13, no. 1: 2020. 36737633 10.1038/s41598-023-27638-9 PMC9898244

[vms370629-bib-0019] Burezq, H. , and F. Khalil . 2025. “Investigating the Impact of Biochar on Methane Gas Emissions and Its Effect on Enteric Fermentation.” Kuwait Journal of Science 52, no. 1: 100332.

[vms370629-bib-0020] Cao, Z. , R. Zhu , Y. Li , et al. 2024. “Mitigation of Ammonia and Hydrogen Sulfide Emissions During Aerobic Composting of Laying Hen Waste Through NaOH‐Modified Biochar.” Journal of Environmental Management 365: 121634. 38943752 10.1016/j.jenvman.2024.121634

[vms370629-bib-0021] Chandrasekharan, S. , S. Jadhav , S. M. Selvam , N. Krishnamoorthy , and P. Balasubramanian . 2024. “Biochar‐Based Materials for Sustainable Energy Applications: A Comprehensive Review.” Journal of Environmental Chemical Engineering 12: 114553.

[vms370629-bib-0022] Chang, H. , C. Zhang , S. Zang , et al. 2024. “Study on the Influencing Mechanism of Biochar and Ethylenediaminetetraacetic Acid Combination on the Remediation of Cd Polluted Soil by *Sedum alfredii* Hance.” Environmental Technology & Innovation 36: 103875.

[vms370629-bib-0222] Chang, J. , D. E. Clay , S. A. Clay , R. Chintala , J. M. Miller , and T. Schumacher . 2016. “Biochar Reduced Nitrous Oxide and Carbon Dioxide Emissions from Soil with Different Water and Temperature Cycles.” Agronomy Journal 108, no. 6: 2214–2221. 10.2134/agronj2016.02.0100.

[vms370629-bib-0023] Chen, B. , D. Zhou , and L. Zhu . 2008. “Transitional Adsorption and Partition of Nonpolar and Polar Aromatic Contaminants by Biochars of Pine Needles With Different Pyrolytic Temperatures.” Environmental Science & Technology 42, no. 14: 5137–5143. 18754360 10.1021/es8002684

[vms370629-bib-0024] Chen, B. , J. A. Koziel , A. Bialowiec , and S. C. O'Brien . 2024. “The Potential Role of Biochar in Mitigating Gaseous Emissions From Livestock Waste – A Mini‐Review.” Journal of Environmental Management 370: 122692. 39401477 10.1016/j.jenvman.2024.122692

[vms370629-bib-0025] Chen, G. , Z. Zhang , Z. Zhang , and R. Zhang . 2018. “Redox‐Active Reactions in Denitrification Provided by Biochars Pyrolyzed at Different Temperatures.” Science of the Total Environment 615: 1547–1556. 28931458 10.1016/j.scitotenv.2017.09.125

[vms370629-bib-0026] Chen, W. , J. Meng , X. Han , Y. Lan , and W. Zhang . 2019. “Past, Present, and Future of Biochar.” Biochar 1, no. 1: 75–87.

[vms370629-bib-0027] Chen, W. , X. Liao , Y. Wu , et al. 2017. “Effects of Different Types of Biochar on Methane and Ammonia Mitigation During Layer Manure Composting.” Waste Management 61: 506–515. 28117129 10.1016/j.wasman.2017.01.014

[vms370629-bib-0028] Chen, X. , S. H. Yang , Z. W. Jiang , J. Ding , and X. Sun . 2021. “Biochar as a Tool to Reduce Environmental Impacts of Nitrogen Loss in Water‐Saving Irrigation Paddy Field.” Journal of Cleaner Production 290: 125811.

[vms370629-bib-0029] Chu, J. , S. Feng , C. Guo , B. Xue , K. He , and L. Li . 2023. “Immunological Mechanisms of Inflammatory Diseases Caused by Gut Microbiota Dysbiosis: A Review.” Biomedicine & Pharmacotherapy 164: 114985. 37311282 10.1016/j.biopha.2023.114985

[vms370629-bib-0030] Chung, W. J. , S. W. Chang , D. K. Chaudhary , et al. 2021. “Effect of Biochar Amendment on Compost Quality, Gaseous Emissions and Pathogen Reduction During In‐Vessel Composting of Chicken Manure.” Chemosphere 283: 131129. 34153920 10.1016/j.chemosphere.2021.131129

[vms370629-bib-0032] Dadrasnia, A. , N. Shahsavari , and C. U. Emenike . 2013. “Remediation of Contaminated Sites.” Hydrocarbon 16: 65–82.

[vms370629-bib-0033] Dai, D. , G. H. Qi , J. Wang , H. J. Zhang , K. Qiu , and S. G. Wu . 2022. “Intestinal Microbiota of Layer Hens and Its Association With Egg Quality and Safety.” Poultry Science 101, no. 9: 102008. 10.1016/j.psj.2022.102008 PMC9289868 35841638

[vms370629-bib-0034] Damiran, D. , K. Larson , and H. Lardner . 2024. “Effect of Biochar Supplementation on Grazing Beef Cow and Calf Performance, Enteric Methane and Carbon Dioxide Emissions, Fecal Egg Counts and Fecal Nutrient Composition.” Sustainable Agriculture Research 13, no. 2: 1–40.

[vms370629-bib-0035] Dansou, D. M. , H. Zhang , Y. Yu , et al. 2023. “Carotenoid Enrichment in Eggs: From Biochemistry Perspective.” Animal Nutrition 14: 315–333. 37635928 10.1016/j.aninu.2023.05.012 PMC10448277

[vms370629-bib-0036] de Oliveira Paiva, I. , E. G. de Morais , K. Jindo , and C. A. Silva . 2024. “Biochar N Content, Pools and Aromaticity as Affected by Feedstock and Pyrolysis Temperature.” Waste and Biomass Valorization 15: 3599–3619.

[vms370629-bib-0037] Dim, C. E. , E. A. Akuru , M. A. Egom , et al. 2018. “Effect of Dietary Inclusion of Biochar on Growth Performance, Haematology and Serum Lipid Profile of Broiler Birds.” Agro‐Science 17, no. 2: 9–17.

[vms370629-bib-0038] Dim, E. C. , E. A. Akuru , F. N. Mgbor , et al. 2022. “Study of Supplementation of Various Levels of Biochar on Health and Production Performance of Growing Local Turkey (*Meleagris gallopova*) Poults.” International Journal of Veterinary Science 11, no. 3: 315–320.

[vms370629-bib-0039] Ding, J. , and S. Yu . 2024. “Impact of Biochar on Nitrogen‐Cycling Functional Genes: A Comparative Study in Mollisol and Alkaline Soils.” Life 14, no. 12: 1631. 39768339 10.3390/life14121631 PMC11677638

[vms370629-bib-0040] Ding, S. , W. Yan , Y. Ma , and J. Fang . 2021. “The Impact of Probiotics on Gut Health via Alternation of Immune Status of Monogastric Animals.” Animal Nutrition 7, no. 1: 24–30. 33997328 10.1016/j.aninu.2020.11.004 PMC8110871

[vms370629-bib-0041] Dittmann, M. T. , C. Baki , M. Terranova , et al. 2024. “The Effect of Biochar Supplementation on Feed Utilization, Milk Production and Methane Emission in Lactating Dairy Cows.” Animal Feed Science and Technology 318: 116127.

[vms370629-bib-0042] Domaradzki, P. , B. Nowakowicz‐Dębek , Ł. Wlazło , M. Ossowski , M. Dmoch , and M. Florek . 2022. “Fatty Acid Composition of Muscle and Adipose Tissue in Pigs Fed With Addition of Natural Sorbents.” Animals 12, no. 13: 1681. 35804580 10.3390/ani12131681 PMC9265011

[vms370629-bib-0043] Dong, J. , P. Jiang , H. Wang , et al. 2023. “Influence of Biomass Feedstocks on Magnetic Biochar Preparation for Efficient Pb(II) Removal.” Environmental Technology & Innovation 32: 103363.

[vms370629-bib-0044] Dong, M. , M. Jiang , L. He , et al. 2025. “Challenges in Safe Environmental Applications of Biochar: Identifying Risks and Unintended Consequence.” Biochar 7, no. 1: 1–20.

[vms370629-bib-0045] Dougherty, B. , M. Gray , M. G. Johnson , and M. Kleber . 2017. “Can Biochar Covers Reduce Emissions From Manure Lagoons While Capturing Nutrients?” Journal of Environmental Quality 46, no. 3: 659–666. 28724092 10.2134/jeq2016.12.0478 PMC7904243

[vms370629-bib-0046] Ebrahimi, M. , S. Gholipour , G. Mostafaii , and F. Yousefian . 2024. “Biochar‐Amended Food Waste Compost: A Review of Properties.” Results in Engineering 24: 103118.

[vms370629-bib-0047] Eckard, R. J. , C. Grainger , and C. A. M. De Klein . 2010. “Options for the Abatement of Methane and Nitrous Oxide From Ruminant Production: A Review.” Livestock Science 130, no. 1–3: 47–56.

[vms370629-bib-0048] Eissa, R. , L. Jeyakumar , D. B. McKenzie , and J. Wu . 2024. “Influence of Biochar Feedstocks on Nitrate Adsorption Capacity.” Earth 5, no. 4: 1080–1096.

[vms370629-bib-0049] Elghalid, O. 2022. “Effect of Graded Levels of Biochar Supplementation as a Growth Promoter on Productive and Physiological Performance of Broiler Chicks.” Egyptian Poultry Science Journal 42, no. 3: 243–263.

[vms370629-bib-0050] Faloye, O. T. , E. A. Ajayi , J. Rostek , et al. 2024. “Hydraulic and Pore Functions of Differently Textured Soils Modified by Biochar From Different Parts of the Mango Plant.” Soil and Tillage Research 236: 105944.

[vms370629-bib-0051] Farghly, M. F. , M. A. Elsagheer , M. M. Jghef , et al. 2023. “Consequences of Supplementing Duck's Diet With Charcoal on Carcass Criteria, Meat Quality, Nutritional Composition, and Bacterial Load.” Poultry Science 102, no. 1: 102275. 10.1016/j.psj.2022.102275 PMC9700026 36427400

[vms370629-bib-0052] Farooq, U. , M. A. Qayyum , and F. Ahmad . 2023. “Effects of Biochar on Poultry: A Review.” Acta Scientific Veterinary Sciences 5, no. 1: 69–73.

[vms370629-bib-0053] Flores, K. R. , A. Fahrenholz , and J. L. Grimes . 2021. “Effect of Pellet Quality and Biochar Litter Amendment on Male Turkey Performance.” Poultry Science 100, no. 4: 101002. 10.1016/j.psj.2021.01.025 PMC7921622 33639349

[vms370629-bib-0054] Forbes, J. D. , G. Van Domselaar , and C. N. Bernstein . 2016. “The Gut Microbiota in Immune‐Mediated Inflammatory Diseases.” Frontiers in Microbiology 7: 1081. 27462309 10.3389/fmicb.2016.01081 PMC4939298

[vms370629-bib-0055] Gao, L. , Z. Dong , Y. Xu , et al. 2025. “Advancements in Biochar Research Methods for Soil Pollution Remediation: Development and Applications.” ACS Omega 10, no. 10: 9854–9868. 40124018 10.1021/acsomega.4c10533 PMC11923846

[vms370629-bib-0056] Glaser, B. , and V. I. Lehr . 2019. “Biochar Effects on Phosphorus Availability in Agricultural Soils: A Meta‐Analysis.” Scientific Reports 9, no. 1: 9338. 31249335 10.1038/s41598-019-45693-z PMC6597700

[vms370629-bib-0057] Goiri, I. , R. Ruiz , R. Atxaerandio , J. L. Lavin , X. D. de Otálora , and A. García‐Rodríguez . 2021. “Assessing the Potential Use of a Feed Additive Based on Biochar on Broilers Feeding Upon Productive Performance, pH of Digestive Organs, Cecum Fermentation and Bacterial Community.” Animal Feed Science and Technology 279: 115039.

[vms370629-bib-0058] Graves, C. , P. Kolar , S. Shah , J. Grimes , and M. Sharara . 2022. “Can Biochar Improve the Sustainability of Animal Production?” Applied Sciences 12, no. 10: 5042.

[vms370629-bib-0059] Gunarathne, V. , S. Mayakaduwa , and M. Vithanage . 2017. “Biochar's Influence as a Soil Amendment for Essential Plant Nutrient Uptake.” In Essential Plant Nutrients: Uptake, Use Efficiency, and Management. Springer.

[vms370629-bib-0060] Guo, L. , H. Yu , W. Niu , and M. Kharbach . 2021. “Biochar Promotes Nitrogen Transformation and Tomato Yield by Regulating Nitrogen‐Related Microorganisms in Tomato Cultivation Soil.” Agronomy 11, no. 2: 381.

[vms370629-bib-0061] Hammerschmiedt, T. , J. Holatko , J. Kucerik , et al. 2022. “Manure Maturation With Biochar: Effects on Plant Biomass, Manure Quality and Soil Microbiological Characteristics.” Agriculture 12, no. 3: 314.

[vms370629-bib-0062] Han, J. , J. Meng , S. Chen , C. Li , and S. Wang . 2018. “Rice Straw Biochar as a Novel Niche for Improved Alterations to the Cecal Microbial Community in Rats.” Scientific Reports 8, no. 1: 16426. 30401962 10.1038/s41598-018-34838-1 PMC6219602

[vms370629-bib-0063] Han, X. , Y. Ma , S. Ding , J. Fang , and G. Liu . 2023. “Regulation of Dietary Fiber on Intestinal Microorganisms and Its Effects on Animal Health.” Animal Nutrition 14: 356–369. 37635930 10.1016/j.aninu.2023.06.004 PMC10448034

[vms370629-bib-0064] Hang, L. T. T. , T. R. Preston , N. X. Ba , and D. Van Dung . 2019. “Effect of Biochar on Growth and Methane Emissions of Goats Fed Fresh Cassava Foliage.” Livestock Research for Rural Development 31, no. 5.

[vms370629-bib-0065] Hasanvand, S. , A. Khatibjoo , H. Shizadi , Y. Mohamadi , M. A. Karimi Torshizi , and D. Rahimhi . 2023. “Effect of Wet Litter Biochar, Probiotic and Zeolite on Performance, Blood Metabolites and Small Intestine Morphology of Broiler Chickens Reared Under Cold Stress.” Animal Production 25, no. 3: 325–341.

[vms370629-bib-0066] Hawryluk‐Sidoruk, M. , M. Raczkiewicz , P. Krasucka , et al. 2024. “Effect of Biochar Chemical Modification (Acid, Base and Hydrogen Peroxide) on Contaminants Content Depending on Feedstock and Pyrolysis Conditions.” Chemical Engineering Journal 481: 148329.

[vms370629-bib-0067] He, D. , Y. Luo , and B. Zhu . 2024. “Feedstock and Pyrolysis Temperature Influence Biochar Properties and Its Interactions With Soil Substances: Insights From a DFT Calculation.” Science of the Total Environment 922: 171259. 38417524 10.1016/j.scitotenv.2024.171259

[vms370629-bib-0068] Hegarty, R. , S. Joseph , M. Rebbeck , S. Meale , and N. Paul . 2024. “Biochar as an Animal Feed Ingredient.” In Biochar for Environmental Management. 3rd ed. edited by J. Lehmann , and S. Joseph , 23. Routledge.

[vms370629-bib-0069] Heinrich, T. , H. Park , R. Orozco , et al. 2023. “Biochar Production From Late‐Harvest Grass – Challenges and Potential for Farm‐Scale Implementation.” Sustainable Production and Consumption 37: 256–267.

[vms370629-bib-0070] Hien, N. N. , N. N. X. Dung , L. H. Manh , and B. T. Le Minh . 2018. “Effects of Biochar Inclusion in Feed and Chicken Litter on Growth Performance, Plasma Lipids and Fecal Bacteria Count of Noi Lai Chicken.” Livestock Research for Rural Development 30, no. 7: 131.

[vms370629-bib-0071] Hilber, I. , A. C. Bastos , S. Loureiro , et al. 2017. “The Different Faces of Biochar: Contamination Risk Versus Remediation Tool.” Journal of Environmental Engineering and Landscape Management 25, no. 2: 86–104.

[vms370629-bib-0072] Hossain, M. Z. , M. M. Bahar , B. Sarkar , et al. 2020. “Biochar and Its Importance on Nutrient Dynamics in Soil and Plant.” Biochar 2, no. 4: 379–420.

[vms370629-bib-0073] Huang, H. , N. G. Reddy , X. Huang , et al. 2021. “Effects of Pyrolysis Temperature, Feedstock Type and Compaction on Water Retention of Biochar Amended Soil.” Scientific Reports 11, no. 1: 7419. 33795757 10.1038/s41598-021-86701-5 PMC8016943

[vms370629-bib-0074] Huang, H. , W. Yao , R. Li , et al. 2018. “Effect of Pyrolysis Temperature on Chemical Form, Behavior and Environmental Risk of Zn, Pb and Cd in Biochar Produced From Phytoremediation Residue.” Bioresource Technology 249: 487–493. 29073559 10.1016/j.biortech.2017.10.020

[vms370629-bib-0075] Huang, X. , and D. U. Ahn . 2019. “Lipid Oxidation and Its Implications to Meat Quality and Human Health.” Food Science and Biotechnology 28: 1275–1285. 31695926 10.1007/s10068-019-00631-7 PMC6811465

[vms370629-bib-0076] Ippolito, J. A. , L. Cui , C. Kammann , et al. 2020. “Feedstock Choice, Pyrolysis Temperature and Type Influence Biochar Characteristics: A Comprehensive Meta‐Data Analysis Review.” Biochar 2: 421–438.

[vms370629-bib-0077] Islam, M. M. , S. T. Ahmed , Y. J. Kim , H. S. Mun , and C. J. Yang . 2014. “Effect of Sea Tangle (*Laminaria japonica*) and Charcoal Supplementation as Alternatives to Antibiotics on Growth Performance and Meat Quality of Ducks.” Asian‐Australasian Journal of Animal Sciences 27: 217–224. 25049946 10.5713/ajas.2013.13314 PMC4093215

[vms370629-bib-0079] Kabir, E. , K. H. Kim , and E. E. Kwon . 2023. “Biochar as a Tool for the Improvement of Soil and Environment.” Frontiers in Environmental Science 11: 1324533.

[vms370629-bib-0080] Kalu, S. , L. Kulmala , J. Zrim , et al. 2022. “Potential of Biochar to Reduce Greenhouse Gas Emissions and Increase Nitrogen Use Efficiency in Boreal Arable Soils in the Long‐Term.” Frontiers in Environmental Science 10: 914766.

[vms370629-bib-0081] Kalus, K. , D. Konkol , M. Korczyński , J. A. Koziel , and S. Opaliński . 2020. “Laying Hens Biochar Diet Supplementation—Effect on Performance, Excreta N Content, NH_3_ and VOCs Emissions, Egg Traits and Egg Consumers Acceptance.” Agriculture 10: 237.

[vms370629-bib-0082] Kammann, C. , J. Ippolito , N. Hagemann , et al. 2017. “Biochar as a Tool to Reduce the Agricultural Greenhouse‐Gas Burden – Knowns, Unknowns and Future Research Needs.” Journal of Environmental Engineering and Landscape Management 25: 114–139.

[vms370629-bib-0083] Kashef, M. , M. Afsharmanesh , and M. Salarmoini . 2021. “Effect of the Substitution of Different Levels of Biochar With Mineral Premix in Diet on Growth Performance Variables, Meat Quality and Bone Ash of Broiler.” Iranian Journal of Animal Science Research 13: 537–549.

[vms370629-bib-0084] Kataya, G. , Z. E. Charif , A. Badran , et al. 2024. “Evaluating the Impact of Different Biochar Types on Wheat Germination.” Scientific Reports 14, no. 1: 28663. 39562575 10.1038/s41598-024-76765-4 PMC11576965

[vms370629-bib-0085] Kavitha, B. , P. V. L. Reddy , B. Kim , S. S. Lee , S. K. Pandey , and K. H. Kim . 2018. “Benefits and Limitations of Biochar Amendment in Agricultural Soils: A Review.” Journal of Environmental Management 227: 146–154. 30176434 10.1016/j.jenvman.2018.08.082

[vms370629-bib-0086] Keba, D. , T. Tolemariam , S. Demeke , and A. Alkhtib . 2023. “Corncob Biochar Supplementation Improves Nutrient Digestibility, Fattening Performance and Carcass Characteristics of Fattening Sheep.” Veterinary Medicine and Science 9: 967–973. 36622266 10.1002/vms3.1072 PMC10029877

[vms370629-bib-0087] Keske, C. , T. Godfrey , D. L. Hoag , and J. Abedin . 2019. “Economic Feasibility of Biochar and Agriculture Coproduction From Canadian Black Spruce Forest.” Food and Energy Security 9: e188.

[vms370629-bib-0088] Khater, E. S. , A. Bahnasawy , R. Hamouda , A. Sabahy , W. Abbas , and O. M. Morsy . 2024. “Biochar Production Under Different Pyrolysis Temperatures With Different Types of Agricultural Wastes.” Scientific Reports 14, no. 1: 2625. 38297102 10.1038/s41598-024-52336-5 PMC10831055

[vms370629-bib-0089] Kim, J. Y. , S. Oh , and Y. K. Park . 2020. “Overview of Biochar Production From Preservative‐Treated Wood With Detailed Analysis of Biochar Characteristics, Heavy Metals Behaviors, and Their Ecotoxicity.” Journal of Hazardous Materials 384: 121356. 31628056 10.1016/j.jhazmat.2019.121356

[vms370629-bib-0090] Kim, K. S. , Y. H. Kim , J. C. Park , et al. 2017. “Effect of Organic Medicinal Charcoal Supplementation in Finishing Pig Diets.” Korean Journal of Agricultural Science 44: 50–59.

[vms370629-bib-0091] Kohira, Y. , S. Akizuki , F. A. Mihretie , D. F. Meselu , S. A. Legesse , and S. Sato . 2024. “Enhancement of Alkali‐ and Oxidation‐Modified Biochars Derived From Water Hyacinth for Ammonium Adsorption Capacity.” Soil Science and Plant Nutrition 70, no. 1: 21–33.

[vms370629-bib-0092] Konduri, A. , V. S. Bharti , S. Krishnan , et al. 2024. “Dietary Biochar Effect on Growth Performance, Proximate Composition, and Physiological Response of *Penaeus vannamei* (Boone, 1931) Cultured in Inland Saline Groundwater.” Animal Feed Science and Technology 316: 116053.

[vms370629-bib-0093] Kumari, K. , R. Kumar , N. Bordoloi , T. Minkina , C. Keswani , and K. Bauddh . 2023. “Unravelling the Recent Developments in the Production Technology and Efficient Applications of Biochar for Agro‐Ecosystems.” Agriculture 13: 512.

[vms370629-bib-0094] Lataf, A. , M. Jozefczak , B. Vandecasteele , et al. 2022. “The Effect of Pyrolysis Temperature and Feedstock on Biochar Agronomic Properties.” Journal of Analytical and Applied Pyrolysis 168: 105728.

[vms370629-bib-0095] Lehmann, J. 2009. “Terra Preta Nova – Where to From Here?.” In Amazonian Dark Earths: Wim Sombroek's Vision. Springer.

[vms370629-bib-0096] Leng, L. , Q. Xiong , L. Yang , et al. 2021. “An Overview on Engineering the Surface Area and Porosity of Biochar.” Science of the Total Environment 763: 144204. 33385838 10.1016/j.scitotenv.2020.144204

[vms370629-bib-0097] Leng, R. A. 2014. “Interactions Between Microbial Consortia in Biofilms: A Paradigm Shift in Rumen Microbial Ecology and Enteric Methane Mitigation.” Animal Production Science 54, no. 5: 519–543.

[vms370629-bib-0098] Leng, R. A. , T. R. Preston , and S. Inthapanya . 2012. “Biochar Reduces Enteric Methane and Improves Growth and Feed Conversion in Local ‘Yellow’ Cattle Fed Cassava Root Chips and Fresh Cassava Foliage.” Livestock Research for Rural Development 24: 199.

[vms370629-bib-0099] Li, H. , Y. Lin , X. Qin , et al. 2024. “An Updated Review on How Biochar May Possess Potential in Soil ARGs Control on Aspects of Source, Fate and Elimination.” Biochar 6: 24.

[vms370629-bib-0100] Li, J. , H. Li , J. Shang , K. Liu , Y. He , and X. Shao . 2023. “The Synergistic Effect of Biochar and Microorganisms Greatly Improves Vegetation and Microbial Structure of Degraded Alpine Grassland on Qinghai–Tibet Plateau.” Agronomy 13: 2203.

[vms370629-bib-0101] Li, J. , Q. Chen , H. Li , et al. 2020. “Impacts of Different Sources of Animal Manures on Dissemination of Human Pathogenic Bacteria in Agricultural Soils.” Environmental Pollution 266: 115399. 32814181 10.1016/j.envpol.2020.115399

[vms370629-bib-0102] Li, Q. , J. Zhang , J. Ye , et al. 2024. “Biochar Affects Organic Carbon Composition and Stability in Highly Acidic Tea Plantation Soil.” Journal of Environmental Management 370: 122803. 39378814 10.1016/j.jenvman.2024.122803

[vms370629-bib-0103] Li, R. , H. Huang , J. J. Wang , et al. 2019. “Conversion of Cu(II)‐Polluted Biomass Into an Environmentally Benign Cu Nanoparticles‐Embedded Biochar Composite and Its Potential Use on Cyanobacteria Inhibition.” Journal of Cleaner Production 216: 25–32.

[vms370629-bib-0104] Li, S. , and D. Tasnady . 2023. “Biochar for Soil Carbon Sequestration: Current Knowledge, Mechanisms, and Future Perspectives.” C—Journal of Carbon Research 9: 67.

[vms370629-bib-0105] Lind, V. , Ö. Sizmaz , A. Demirtas , et al. 2024. “Biochar Effect on Sheep Feed Intake, Growth Rate and Ruminant In Vitro and In Vivo Methane Production.” Animal 18: 101195. 38850574 10.1016/j.animal.2024.101195

[vms370629-bib-0106] Liu, D. , W. Zhang , H. Lin , Y. Li , H. Lu , and Y. Wang . 2016. “A Green Technology for the Preparation of High Capacitance Rice Husk‐Based Activated Carbon.” Journal of Cleaner Production 112: 1190–1198.

[vms370629-bib-0107] Liu, D. , Z. Feng , H. Zhu , et al. 2020. “Effects of Corn Straw Biochar Application on Soybean Growth and Alkaline Soil Properties.” BioResources 15, no. 1: 1463–1481.

[vms370629-bib-0108] Liu, J. , S. Q. Han , J. H. Qi , and Q. Zhou . 2016. “Influence of Biochar Content on In‐Situ Denitrification of Sediment and Microbial Activity.” Jiangsu Agricultural Journal 32: 106–110.

[vms370629-bib-0109] Liu, Z. , T. He , T. Cao , T. Yang , J. Meng , and W. Chen . 2017. “Effects of Biochar Application on Nitrogen Leaching, Ammonia Volatilization and Nitrogen Use Efficiency in Two Distinct Soils.” Journal of Soil Science and Plant Nutrition 17, no. 2: 515–528.

[vms370629-bib-0110] Lorenz, K. , and R. Lal . 2014. “Biochar Application to Soil for Climate Change Mitigation by Soil Organic Carbon Sequestration.” Journal of Plant Nutrition and Soil Science 177: 651–670.

[vms370629-bib-0111] Luo, J. , L. Lin , C. Liu , et al. 2020. “Reveal a Hidden Highly Toxic Substance in Biochar to Support Its Effective Elimination Strategy.” Journal of Hazardous Materials 399: 123055. 32526445 10.1016/j.jhazmat.2020.123055

[vms370629-bib-0113] Martin, L. B. , E. Andreassi , W. Watson , and C. Coon . 2011. “Stress and Animal Health: Physiological Mechanisms and Ecological Consequences.” Nature Education Knowledge 3: 11.

[vms370629-bib-0114] Martinez‐Fernandez, G. , R. D. Kinley , W. J. Smith , et al. 2024. “Effect of Fit‐for‐Purpose Biochars on Rumen Fermentation, Microbial Communities, and Methane Production in Cattle.” Frontiers in Microbiology 15: 1463817. 39629207 10.3389/fmicb.2024.1463817 PMC11611548

[vms370629-bib-0115] McAvoy, D. J. , B. Burritt , and J. J. Villalba . 2020. “Use of Biochar by Sheep: Impacts on Diet Selection, Digestibility, and Performance.” Journal of Animal Science 98: skaa380. 33221902 10.1093/jas/skaa380 PMC7755174

[vms370629-bib-0116] Mirheidari, A. , N. M. Torbatinejad , P. Shakeri , and A. Mokhtarpour . 2020. “Effects of Biochar Produced From Different Biomass Sources on Digestibility, Ruminal Fermentation, Microbial Protein Synthesis and Growth Performance of Male Lambs.” Small Ruminant Research 183: 106042.

[vms370629-bib-0117] Mirheidari, A. , N. M. Torbatinejad , P. Shakeri , and A. Mokhtarpour . 2019. “Effects of Walnut Shell and Chicken Manure Biochar on In Vitro Fermentation and In Vivo Nutrient Digestibility and Performance of Dairy Ewes.” Tropical Animal Health and Production 51: 2153–2160. 31079336 10.1007/s11250-019-01909-y

[vms370629-bib-0118] Mohammadi, A. 2021. “Overview of the Benefits and Challenges Associated With Pelletizing Biochar.” Processes 9: 1591.

[vms370629-bib-0119] Mukherjee, D. , M. Sil , A. Goswami , et al. 2025. “Synthesis, Modification and Antimicrobial Potential of Biochar and Its Modifications Against Water‐Borne Pathogens: A Review.” Results in Surfaces and Interfaces 18: 100438.

[vms370629-bib-0120] Mukherjee, S. , B. Sarkar , V. K. Aralappanavar , et al. 2022. “Biochar‐Microorganism Interactions for Organic Pollutant Remediation: Challenges and Perspectives.” Environmental Pollution 308: 119609. 35700879 10.1016/j.envpol.2022.119609

[vms370629-bib-0122] Musa, N. , K. S. Khan , J. C. Blankinship , et al. 2024. “Sorption‐Desorption of Phosphorus on Manure‐ and Plant‐Derived Biochars at Different Pyrolysis Temperatures.” Sustainability 16, no. 7: 2755.

[vms370629-bib-0123] Muzolf‐Panek, M. , A. Zaworska‐Zakrzewska , A. Czech , D. Lisiak , and M. Kasprowicz‐Potocka . 2024. “Antioxidative Status and Meat Quality Traits as Affected by Dietary Supplementation of Finishing Pigs With Natural Phenolics.” Antioxidants 13: 1362. 39594504 10.3390/antiox13111362 PMC11590991

[vms370629-bib-0124] Myung, E. , H. Kim , N. Choi , and K. Cho . 2024. “The Biochar Derived From Spirulina Platensis for the Adsorption of Pb and Zn and Enhancing the Soil Physicochemical Properties.” Chemosphere 364: 143203. 39209036 10.1016/j.chemosphere.2024.143203

[vms370629-bib-0125] Naeem, M. , and D. Bourassa . 2025. “Probiotics in Poultry: Unlocking Productivity Through Microbiome Modulation and Gut Health.” Microorganisms 13: 257. 40005624 10.3390/microorganisms13020257 PMC11857632

[vms370629-bib-0126] Nair, P. S. , M. P. Sivani , S. Suresh , et al. 2023. “Beneficial Impacts of Biochar as a Potential Feed Additive in Animal Husbandry.” Journal of Experimental Biology and Agricultural Sciences 11: 479–499.

[vms370629-bib-0127] Nayak, N. , R. Mehrotra , and S. Mehrotra . 2022. “Carbon Biosequestration Strategies: A Review.” Carbon Capture Science & Technology 4: 100065.

[vms370629-bib-0128] Ndirangu, S. M. , Y. Liu , K. Xu , and S. Song . 2019. “Risk Evaluation of Pyrolyzed Biochar From Multiple Wastes.” Journal of Chemistry 2019: 4506314.

[vms370629-bib-0129] Nepal, J. , W. Ahmad , F. Munsif , A. Khan , and Z. Zou . 2023. “Advances and Prospects of Biochar in Improving Soil Fertility, Biochemical Quality, and Environmental Applications.” Frontiers in Environmental Science 11: 1114752.

[vms370629-bib-0130] Nguyen, B. T. , J. Lehmann , W. C. Hockaday , S. Joseph , and C. A. Masiello . 2010. “Temperature Sensitivity of Black Carbon Decomposition and Oxidation.” Environmental Science & Technology 44: 3324–3331. 20384335 10.1021/es903016y

[vms370629-bib-0131] Nguyen, T. B. , Q. H. Do , C. W. Chen , W. H. Chen , X. T. Bui , and C. D. Dong . 2024. “Pyrolysis Temperature Effect on Biochar‐Derived Cow Manure: Physicochemical Properties and Adsorption Behavior Toward Organic Dyes.” Journal of the Taiwan Institute of Chemical Engineers 164: 105675.

[vms370629-bib-0132] Norton, T. , C. Chen , M. L. V. Larsen , and D. Berckmans . 2019. “Precision Livestock Farming: Building ‘Digital Representations’ to Bring the Animals Closer to the Farmer.” Animal 13: 3009–3017. 31516101 10.1017/S175173111900199X

[vms370629-bib-0133] Nosratabad, N. A. , Q. Yan , Z. Cai , and C. Wan . 2024. “Exploring Nanomaterial‐Modified Biochar for Environmental Remediation Applications.” Heliyon 10: e37123. 39315228 10.1016/j.heliyon.2024.e37123 PMC11417198

[vms370629-bib-0134] O'Toole, A. , D. Andersson , A. Gerlach , et al. 2016. “Current and Future Applications for Biochar.” In Biochar in European Soils and Agriculture. Routledge.

[vms370629-bib-0135] Osman, A. I. , S. Fawzy , M. Farghali , et al. 2022. “Biochar for Agronomy, Animal Farming, Anaerobic Digestion, Composting, Water Treatment, Soil Remediation, Construction, Energy Storage, and Carbon Sequestration: A Review.” Environmental Chemistry Letters 20: 2385–2485. 35571983 10.1007/s10311-022-01424-x PMC9077033

[vms370629-bib-0136] Panchal, J. , A. Patel , S. Patel , and D. Goswami . 2024. “Understanding Mastitis: Microbiome, Control Strategies, and Prevalence – A Comprehensive Review.” Microbial Pathogenesis 187: 106533. 38171428 10.1016/j.micpath.2023.106533

[vms370629-bib-0137] Pandian, K. , S. Vijayakumar , M. R. A. F. Mustaffa , P. Subramanian , and S. Chitraputhirapillai . 2024. “Biochar – A Sustainable Soil Conditioner for Improving Soil Health, Crop Production and Environment Under Changing Climate: A Review.” Frontiers in Soil Science 4: 1376159.

[vms370629-bib-0138] Panwar, R. B. , R. P. Sequeira , and T. B. Clarke . 2021. “Microbiota‐Mediated Protection Against Antibiotic‐Resistant Pathogens.” Genes & Immunity 22: 255–267. 33947987 10.1038/s41435-021-00129-5 PMC8497270

[vms370629-bib-0139] Patel, M. , A. O. Oyedun , A. Kumar , and J. Doucette . 2019. “The Development of a Cost Model for Two Supply Chain Network Scenarios for Decentralized Pyrolysis System Scenarios to Produce Bio‐Oil.” Biomass and Bioenergy 128: 105287.

[vms370629-bib-0140] Pavesi, R. , L. Orsi , and L. Zanderighi . 2025. “Enhancing Circularity in Urban Waste Management: A Case Study on Biochar From Urban Pruning.” Environments 12: 1–21.

[vms370629-bib-0141] Pinelli, S. , S. Rossi , A. Malcevschi , et al. 2024. “Biochar Dust Emission: Is It a Health Concern? Preliminary Results for Toxicity Assessment.” Environmental Toxicology and Pharmacology 109: 104477. 38810713 10.1016/j.etap.2024.104477

[vms370629-bib-0142] Prasad, M. , A. Chrysargyris , N. McDaniel , A. Kavanagh , N. S. Gruda , and N. Tzortzakis . 2019. “Plant Nutrient Availability and pH of Biochars and Their Fractions, With the Possible Use as a Component in a Growing Media.” Agronomy 10: 10.

[vms370629-bib-0143] Prasai, T. P. , K. B. Walsh , D. J. Midmore , and S. P. Bhattarai . 2017. “Effect of Biochar, Zeolite and Bentonite Feed Supplements on Egg Yield and Excreta Attributes.” Animal Production Science 58, no. 9: 1632–1641.

[vms370629-bib-0144] Prasai, T. P. , K. B. Walsh , D. J. Midmore , B. E. H. Jones , and S. P. Bhattarai . 2018. “Manure From Biochar, Bentonite and Zeolite Feed Supplemented Poultry: Moisture Retention and Granulation Properties.” Journal of Environmental Management 216: 82–88. 28867404 10.1016/j.jenvman.2017.08.040

[vms370629-bib-0145] Prasai, T. P. , K. B. Walsh , S. P. Bhattarai , et al. 2016. “Biochar, Bentonite and Zeolite Supplemented Feeding of Layer Chickens Alters Intestinal Microbiota and Reduces Campylobacter Load.” PLoS ONE 11, no. 4: e0154061. 27116607 10.1371/journal.pone.0154061 PMC4845986

[vms370629-bib-0146] Prévoteau, A. , F. Ronsse , I. Cid , P. Boeckx , and K. Rabaey . 2016. “The Electron Donating Capacity of Biochar Is Dramatically Underestimated.” Scientific Reports 6, no. 1: 32870. 27628746 10.1038/srep32870 PMC5024093

[vms370629-bib-0147] Raina, N. , M. Zavalloni , and D. Viaggi . 2024. “Incentive Mechanisms of Carbon Farming Contracts: A Systematic Mapping Study.” Journal of Environmental Management 352: 120126. 38271871 10.1016/j.jenvman.2024.120126

[vms370629-bib-0148] Rashidi, N. , A. Khatibjoo , K. Taherpour , M. Akbari‐Gharaei , and H. Shirzadi . 2020. “Effects of Licorice Extract, Probiotic, Toxin Binder and Poultry Litter Biochar on Performance, Immune Function, Blood Indices and Liver Histopathology of Broilers Exposed to Aflatoxin‐B1.” Poultry Science 99, no. 11: 5896–5906. 10.1016/j.psj.2020.08.034 PMC7647870 33142507

[vms370629-bib-0149] Ravindran, B. , H. A. Mupambwa , S. Silwana , and P. N. Mnkeni . 2017. “Assessment of Nutrient Quality, Heavy Metals and Phytotoxic Properties of Chicken Manure on Selected Commercial Vegetable Crops.” Heliyon 3, no. 12: e00493. 29326987 10.1016/j.heliyon.2017.e00493 PMC5760451

[vms370629-bib-0150] Reggi, S. , S. Frazzini , S. Pedrazzi , et al. 2025. “Metabolomic Insights Into the Potential of Chestnut Biochar as a Functional Feed Ingredient.” Applied Sciences 15, no. 3: 1084.

[vms370629-bib-0151] Rehman, A. , S. Nawaz , H. A. Alghamdi , S. Alrumman , W. Yan , and M. Z. Nawaz . 2020. “Effects of Manure‐Based Biochar on Uptake of Nutrients and Water Holding Capacity of Different Types of Soils.” Case Studies in Chemical and Environmental Engineering 2: 100036.

[vms370629-bib-0152] Rogovska, N. , D. Laird , R. Cruse , P. Fleming , T. Parkin , and D. Meek . 2011. “Impact of Biochar on Manure Carbon Stabilization and Greenhouse Gas Emissions.” Soil Science Society of America Journal 75, no. 3: 871–879.

[vms370629-bib-0153] Rosman, N. Z. I. B. , and N. S. Jamaludin . 2023. “Biochar and Chicken Manure Compost.” In Handbook of Biodegradable Materials, edited by G. A. M. Ali and A. S. H. Makhlouf , 1–15. Springer. 10.1007/978-3-031-09710-2_51.

[vms370629-bib-0154] Saeidi Garaghani, S. , M. Bashtani , P. Shakeri , and H. Naeimipour Younesi . 2023. “Effect of Mineral Biochar Feeding on Growth Performance, Nutrient Digestibility, Blood and Fermentation Parameters of Weaned Holstein Calves.” Journal of Ruminant Research 10, no. 4: 121–136.

[vms370629-bib-0155] Sajjadi, B. , T. Zubatiuk , D. Leszczynska , J. Leszczynski , and W. Y. Chen . 2019. “Chemical Activation of Biochar for Energy and Environmental Applications: A Comprehensive Review.” Reviews in Chemical Engineering 35, no. 7: 777–815.

[vms370629-bib-0156] Sánchez‐Monedero, M. A. , M. Sánchez‐García , J. A. Alburquerque , and M. L. Cayuela . 2019. “Biochar Reduces Volatile Organic Compounds Generated During Chicken Manure Composting.” Bioresource Technology 288: 121584. 31178262 10.1016/j.biortech.2019.121584

[vms370629-bib-0157] Satari, B. , J. Khazaei , and M. H. Kianmehr . 2025. “Integrated Hydrothermal Carbonization to Enhance Resource and Energy Recovery From Food Waste.” Applied Food Research 5, no. 1: 100869.

[vms370629-bib-0158] Schmidt, H. P. , N. Hagemann , K. Draper , and C. Kammann . 2019. “The Use of Biochar in Animal Feeding.” PeerJ 7: e7373. 31396445 10.7717/peerj.7373 PMC6679646

[vms370629-bib-0159] Selvaraj, P. S. , P. Ettiyagounder , K. Sabarish , et al. 2025. “Hydrothermal Carbonization Approach for Transforming Biomass Waste to Value Added Hydrochar and Its Applications in Water Remediation.” Desalination and Water Treatment 322: 101199.

[vms370629-bib-0160] Seow, Y. X. , Y. H. Tan , N. M. Mubarak , et al. 2022. “A Review on Biochar Production From Different Biomass Wastes by Recent Carbonization Technologies and Its Sustainable Applications.” Journal of Environmental Chemical Engineering 10, no. 1: 107017.

[vms370629-bib-0161] Sethupathi, S. , M. Zhang , A. U. Rajapaksha , et al. 2017. “Biochars as Potential Adsorbers of CH_4_, CO_2_ and H_2_S.” Sustainability 9, no. 1: 121.

[vms370629-bib-0162] Shankar, A. H. , and A. S. Prasad . 1998. “Zinc and Immune Function: The Biological Basis of Altered Resistance to Infection.” American Journal of Clinical Nutrition 68, no. 2: 447S–463S. 9701160 10.1093/ajcn/68.2.447S

[vms370629-bib-0163] Shoudho, K. N. , T. H. Khan , U. R. Ara , M. R. Khan , Z. B. Z. Shawon , and M. E. Hoque . 2024. “Biochar in Global Carbon Cycle: Towards Sustainable Development Goals.” Current Research in Green and Sustainable Chemistry 8: 100409.

[vms370629-bib-0164] Shrestha, R. K. , P. A. Jacinthe , R. Lal , et al. 2023. “Biochar as a Negative Emission Technology: A Synthesis of Field Research on Greenhouse Gas Emissions.” Journal of Environmental Quality 52, no. 4: 769–798. 36905388 10.1002/jeq2.20475

[vms370629-bib-0165] Silivong, P. , and T. R. Preston . 2015. “Growth Performance of Goats Was Improved When a Basal Diet of Foliage of Bauhinia Acuminata Was Supplemented With Water Spinach and Biochar.” Livestock Research for Rural Development 27, no. 3: 58.

[vms370629-bib-0166] Singh, H. P. , P. Mahajan , S. Kaur , D. R. Batish , and R. K. Kohli . 2013. “Chromium Toxicity and Tolerance in Plants.” Environmental Chemistry Letters 11: 229–254.

[vms370629-bib-0167] Sirjani, M. H. , J. Rezaei , M. Zahedifar , and Y. Rouzbehan . 2023. “Effect of Adding Biochar in Diets Containing Probiotics on In Vitro Fermentation Variables, Health Indicators, Rectum Bacteria, and Blood Enzymes of Holstein Calves.” Animal Production Research 11, no. 4: 1–19.

[vms370629-bib-0168] Sobol, Ł. , and A. Dyjakon . 2022. “Biochar as a Sustainable Product for the Removal of Odor Emissions—Mini Literature Review.” Revista De Chimie 73, no. 4: 86–96.

[vms370629-bib-0169] Song, J. , Y. Wang , S. Zhang , et al. 2021. “Coupling Biochar With Anaerobic Digestion in a Circular Economy Perspective: A Promising Way to Promote Sustainable Energy, Environment and Agriculture Development in China.” Renewable and Sustainable Energy Reviews 144: 110973.

[vms370629-bib-0170] Sperber, J. L. , B. C. Troyer , G. E. Erickson , and A. K. Watson . 2022. “Evaluation of the Effects of Pine‐Sourced Biochar on Cattle Performance and Methane and Carbon Dioxide Production From Growing and Finishing Steers.” Translational Animal Science 6, no. 4: txac152. 36568901 10.1093/tas/txac152 PMC9769114

[vms370629-bib-0171] Sri Shalini, S. , K. Palanivelu , A. Ramachandran , and V. Raghavan . 2021. “Biochar From Biomass Waste as a Renewable Carbon Material for Climate Change Mitigation in Reducing Greenhouse Gas Emissions—A Review.” Biomass Conversion and Biorefinery 11, no. 5: 2247–2267.

[vms370629-bib-0172] Suliman, W. , J. B. Harsh , N. I. Abu‐Lail , A. M. Fortuna , I. Dallmeyer , and M. Garcia‐Perez . 2016. “Influence of Feedstock Source and Pyrolysis Temperature on Biochar Bulk and Surface Properties.” Biomass and Bioenergy 84: 37–48.

[vms370629-bib-0173] Tahery, S. , M. Rebbeck , S. Joseph , et al. 2023. “Overall Benefits of Biochar, Fed to Dairy Cows, for the Farming System.” Pedosphere 33, no. 1: 225–230.

[vms370629-bib-0174] Tamayao, P. J. , G. O. Ribeiro , T. A. McAllister , et al. 2021. “Effects of Post‐Pyrolysis Treated Biochars on Methane Production, Ruminal Fermentation, and Rumen Microbiota of a Silage‐Based Diet in an Artificial Rumen System (RUSITEC).” Animal Feed Science and Technology 273: 114802.

[vms370629-bib-0175] Tamayao, P. , G. O. Ribeiro , T. A. McAllister , K. H. Ominski , E. K. Okine , and E. J. McGeough . 2022. “Effects of Biochar Source, Level of Inclusion, and Particle Size on In Vitro Dry Matter Disappearance, Total Gas, and Methane Production and Ruminal Fermentation Parameters in a Barley Silage‐Based Diet.” Canadian Journal of Animal Science 102, no. 1: 133–144.

[vms370629-bib-0176] Tan, X. F. , S. S. Zhu , R. P. Wang , et al. 2021. “Role of Biochar Surface Characteristics in the Adsorption of Aromatic Compounds: Pore Structure and Functional Groups.” Chinese Chemical Letters 32, no. 10: 2939–2946.

[vms370629-bib-0177] Tang, J. , Z. Hu , H. Yang , et al. 2024. “Enhancement of Caproate Production From Food Waste Using Biochar Produced From Agricultural Wastes: A Microbial Perspective.” Industrial Crops and Products 221: 119401.

[vms370629-bib-0178] Teoh, R. , E. Caro , D. B. Holman , S. Joseph , S. J. Meale , and A. V. Chaves . 2019. “Effects of Hardwood Biochar on Methane Production, Fermentation Characteristics, and the Rumen Microbiota Using Rumen Simulation.” Frontiers in Microbiology 10: 1534. 31354652 10.3389/fmicb.2019.01534 PMC6635593

[vms370629-bib-0179] Terler, G. , M. Winter , M. Mandl , J. Sweeney , and A. Steinwidder . 2023. “Effect of Biochar or Biochar and Urea Supplementation on Feed Intake, Milk Yield, Feed Conversion and Methane Production of Dairy Cows.” Czech Journal of Animal Science 68, no. 6: 245–254.

[vms370629-bib-0180] Terry, S. A. , A. A. P. Redman , G. O. Ribeiro , et al. 2020. “Effect of a Pine Enhanced Biochar on Growth Performance, Carcass Quality, and Feeding Behavior of Feedlot Steers.” Translational Animal Science 4, no. 2: 831–838. 32734143 10.1093/tas/txaa011 PMC7201079

[vms370629-bib-0181] Thomas, S. C. 2021. “Post‐Processing of Biochars to Enhance Plant Growth Responses: A Review and Meta‐Analysis.” Biochar 3, no. 4: 437–455. 34723131 10.1007/s42773-021-00115-0 PMC8547209

[vms370629-bib-0182] Tomczyk, A. , Z. Sokołowska , and P. Boguta . 2020. “Biochar Physicochemical Properties: Pyrolysis Temperature and Feedstock Kind Effects.” Reviews in Environmental Science and Bio/Technology 19, no. 1: 191–215.

[vms370629-bib-0183] Toth, J. D. , and Z. Dou . 2016. “Use and Impact of Biochar and Charcoal in Animal Production Systems.” Agricultural and Environmental Applications of Biochar: Advances and Barriers 63: 199–224.

[vms370629-bib-0184] Tseten, T. , R. A. Sanjorjo , M. Kwon , and S. W. Kim . 2022. “Strategies to Mitigate Enteric Methane Emissions From Ruminant Animals.” Journal of Microbiology and Biotechnology 32, no. 3: 269–277. 35283433 10.4014/jmb.2202.02019 PMC9628856

[vms370629-bib-0185] Tsolis, V. , and P. Barouchas . 2023. “Biochar as Soil Amendment: The Effect of Biochar on Soil Properties Using VIS‐NIR Diffuse Reflectance Spectroscopy, Biochar Aging and Soil Microbiology—A Review.” Land 12, no. 8: 1580.

[vms370629-bib-0186] Velichkova, R. , I. Simova , M. Pushkarov , and A. Stanilovd . 2024. “A Sustainable Approach to Improve Soil Health. A Case Study for Western Bulgaria.” In 2024 XXXIII International Scientific Conference Electronics (ET) . IEEE.

[vms370629-bib-0187] Viaene, J. , N. Peiren , D. Vandamme , et al. 2024. “Application of Biochar to Anaerobic Digestion Versus Digestate: Effects on N Emissions and C Stability.” Science of the Total Environment 915: 170124. 38232844 10.1016/j.scitotenv.2024.170124

[vms370629-bib-0188] Vijayaraghavan, K. 2021. “The Importance of Mineral Ingredients in Biochar Production, Properties and Applications.” Critical Reviews in Environmental Science and Technology 51, no. 2: 113–139.

[vms370629-bib-0189] Vlasova, A. N. , and L. J. Saif . 2021. “Bovine Immunology: Implications for Dairy Cattle.” Frontiers in Immunology 12: 643206. 34267745 10.3389/fimmu.2021.643206 PMC8276037

[vms370629-bib-0190] Waheed, A. , H. Xu , X. Qiao , et al. 2025. “Biochar in Sustainable Agriculture and Climate Mitigation: Mechanisms, Challenges, and Applications in the Circular Bioeconomy.” Biomass and Bioenergy 193: 107531.

[vms370629-bib-0191] Wang, J. , and S. Wang . 2019. “Preparation, Modification and Environmental Application of Biochar: A Review.” Journal of Cleaner Production 227: 1002–1022.

[vms370629-bib-0192] Wang, J. , Z. Zhao , and Y. Zhang . 2021. “Enhancing Anaerobic Digestion of Kitchen Wastes With Biochar: Link Between Different Properties and Critical Mechanisms of Promoting Interspecies Electron Transfer.” Renewable Energy 167: 791–799.

[vms370629-bib-0193] Wang, N. , Z. Z. Chang , X. M. Xue , et al. 2017. “Biochar Decreases Nitrogen Oxide and Enhances Methane Emissions via Altering Microbial Community Composition of Anaerobic Paddy Soil.” Science of the Total Environment 581: 689–696. 28063654 10.1016/j.scitotenv.2016.12.181

[vms370629-bib-0194] Wang, W. , J. Bai , Q. Lu , et al. 2021. “Pyrolysis Temperature and Feedstock Alter the Functional Groups and Carbon Sequestration Potential of *Phragmites australis*‐ and *Spartina alterniflora*‐Derived Biochars.” GCB Bioenergy 13, no. 3: 493–506.

[vms370629-bib-0195] Wang, Y. , H. Li , and S. Lin . 2022. “Advances in the Study of Heavy Metal Adsorption From Water and Soil by Modified Biochar.” Water 14, no. 23: 3894.

[vms370629-bib-0196] Wang, Y. , K. Wang , X. Wang , Q. Zhao , J. Jiang , and M. Jiang . 2024. “Effect of Different Production Methods on Physicochemical Properties and Adsorption Capacities of Biochar From Sewage Sludge and Kitchen Waste: Mechanism and Correlation Analysis.” Journal of Hazardous Materials 461: 132690. 37801977 10.1016/j.jhazmat.2023.132690

[vms370629-bib-0197] Wen, C. , T. Liu , D. Wang , et al. 2023. “Biochar as the Effective Adsorbent to Combustion Gaseous Pollutants: Preparation, Activation, Functionalization and the Adsorption Mechanisms.” Progress in Energy and Combustion Science 99: 101098.

[vms370629-bib-0198] Willson, N. L. , T. T. Van , S. P. Bhattarai , et al. 2019. “Feed Supplementation With Biochar May Reduce Poultry Pathogens, Including Campylobacter Hepaticus, the Causative Agent of Spotty Liver Disease.” PLoS ONE 14, no. 4: e0214471. 30943226 10.1371/journal.pone.0214471 PMC6447184

[vms370629-bib-0199] Winders, T. M. , M. L. Jolly‐Breithaupt , H. C. Wilson , J. C. MacDonald , G. E. Erickson , and A. K. Watson . 2019. “Evaluation of the Effects of Biochar on Diet Digestibility and Methane Production From Growing and Finishing Steers.” Translational Animal Science 3, no. 2: 775–783. 32704845 10.1093/tas/txz027 PMC7200811

[vms370629-bib-0200] Won, S. , N. Ahmed , B. G. You , S. Shim , S. S. Kim , and C. Ra . 2018. “Nutrient Production From Korean Poultry and Loading Estimations for Cropland.” Journal of Animal Science and Technology 60: 1–9. 29479455 10.1186/s40781-018-0160-1 PMC5817801

[vms370629-bib-0201] Wu, Z. F. , Z. K. Wang , J. B. Li , et al. 2024. “Effects of Biochars Derived From Different Feedstocks and Pyrolysis Temperatures on the Anaerobic Digestion of Kitchen Waste.” Renewable Energy 230: 120833.

[vms370629-bib-0202] Xiang, W. , X. Zhang , J. Chen , et al. 2020. “Biochar Technology in Wastewater Treatment: A Critical Review.” Chemosphere 252: 126539. 32220719 10.1016/j.chemosphere.2020.126539

[vms370629-bib-0203] Xiu, L. , W. Zhang , D. Wu , et al. 2021. “Biochar Can Improve Biological Nitrogen Fixation by Altering the Root Growth Strategy of Soybean in Albic Soil.” Science of the Total Environment 773: 144564. 33940700 10.1016/j.scitotenv.2020.144564

[vms370629-bib-0204] Yaashikaa, P. R. , P. S. Kumar , S. Varjani , and A. J. B. R. Saravanan . 2020. “A Critical Review on the Biochar Production Techniques, Characterization, Stability and Applications for Circular Bioeconomy.” Biotechnology Reports 28: e00570. 33304842 10.1016/j.btre.2020.e00570 PMC7718465

[vms370629-bib-0205] Yadav, A. , P. Yadav , S. Bojjagani , J. K. Srivastava , and A. Raj . 2024. “Investigation of the Speciation and Environmental Risk of Heavy Metals in Biochar Produced From Textile Sludge Waste by Pyrolysis at Different Temperatures.” Chemosphere 360: 142454. 38810801 10.1016/j.chemosphere.2024.142454

[vms370629-bib-0206] Yadav, S. P. S. , S. Bhandari , D. Bhatta , et al. 2023. “Biochar Application: A Sustainable Approach to Improve Soil Health.” Journal of Agriculture and Food Research 11: 100498.

[vms370629-bib-0207] Yan, Q. , N. A. Nosratabad , X. Du , T. Ketelboeter , C. Wan , and Z. Cai . 2025. “Highly Effective Lead Removal by Novel Alkaline Biochar Prepared by Pyrolysis of Woody Biomass Impregnated With Low‐Level NaOH.” Journal of Hazardous Materials Advances 18: 100657.

[vms370629-bib-0208] Ye, S. , B. R. Shah , J. Li , et al. 2022. “A Critical Review on Interplay Between Dietary Fibers and Gut Microbiota.” Trends in Food Science & Technology 124: 237–249.

[vms370629-bib-0209] Yıldızlı, G. , G. Coral , and F. Ayaz . 2021. “Biochar as a Biocompatible Mild Anti‐Inflammatory Supplement for Animal Feed and Agricultural Fields.” Chemistry & Biodiversity 18, no. 6: e2001002. 33835673 10.1002/cbdv.202001002

[vms370629-bib-0210] Yoo, J. Y. , M. Groer , S. V. O. Dutra , A. Sarkar , and D. I. McSkimming . 2020. “Gut Microbiota and Immune System Interactions.” Microorganisms 8, no. 10: 1587. 33076307 10.3390/microorganisms8101587 PMC7602490

[vms370629-bib-0211] Zbair, M. , M. Drané , and L. Limousy . 2024. “NO_2_ Adsorption on Biochar Derived From Wood Shaving Litter: Understanding Surface Chemistry and Adsorption Mechanisms.” Clean Technologies 6, no. 3: 973–993.

[vms370629-bib-0212] Zhang, F. , X. Li , and Y. Wei . 2023. “Selenium and Selenoproteins in Health.” Biomolecules 13, no. 5: 799. 37238669 10.3390/biom13050799 PMC10216560

[vms370629-bib-0213] Zhang, H. , J. N. Marchant‐Forde , X. Zhang , and Y. Wang . 2020. “Effect of Cornstalk Biochar Immobilized Bacteria on Ammonia Reduction in Laying Hen Manure Composting.” Molecules (Basel, Switzerland) 25, no. 7: 1560. 32231157 10.3390/molecules25071560 PMC7181132

[vms370629-bib-0214] Zhang, L. , Z. Yao , L. Zhao , et al. 2024. “Effects of Various Pyrolysis Temperatures on the Physicochemical Characteristics of Crop Straw‐Derived Biochars and Their Application in Tar Reforming.” Catalysis Today 433: 114663.

[vms370629-bib-0215] Zhang, P. , W. Duan , H. Peng , B. Pan , and B. Xing . 2021. “Functional Biochar and Its Balanced Design.” ACS Environmental Au 2, no. 2: 115–127. 37101585 10.1021/acsenvironau.1c00032 PMC10114722

[vms370629-bib-0216] Zhang, W. , J. Shen , H. Zhang , et al. 2021. “Efficient Nitrate Removal by Pseudomonas Mendocina GL6 Immobilized on Biochar.” Bioresource Technology 320: 124324. 33147528 10.1016/j.biortech.2020.124324

[vms370629-bib-0217] Zhao, Q. , J. Cui , Y. Hou , and P. Pei . 2024. “Effect of Pyrolysis Temperature on Physicochemical Characteristics and Toxic Elements for Grub Manure‐Derived Biochar.” RSC Advances 14, no. 38: 27883–27893. 39224651 10.1039/d4ra03778b PMC11367629

[vms370629-bib-0218] Zhou, J. B. , M. M. Jiang , and G. Q. Chen . 2007. “Estimation of Methane and Nitrous Oxide Emission From Livestock and Poultry in China During 1949–2003.” Energy Policy 35, no. 7: 3759–3767.

[vms370629-bib-0219] Zhou, Y. , S. Wang , and L. Chen . 2025. “Progress and Prospects of Biochar as Concrete Filler: A Review.” Alexandria Engineering Journal 128: 306–323.

[vms370629-bib-0220] Zhu, B. , B. Wan , T. Liu , et al. 2023. “Biochar Enhances Multifunctionality by Increasing the Uniformity of Energy Flow Through a Soil Nematode Food Web.” Soil Biology and Biochemistry 183: 109056.

[vms370629-bib-0221] Zhu, Z. , Y. Zhang , W. Tao , X. Zhang , Z. Xu , and C. Xu . 2025. “The Biological Effects of Biochar on Soil's Physical and Chemical Characteristics: A Review.” Sustainability 17, no. 5: 2214.

